# Targeting Glycolytic Metabolism in Cancer Therapy: Current Approaches and Future Perspectives

**DOI:** 10.3390/cells15040362

**Published:** 2026-02-18

**Authors:** Shuang Li, Jie Gong, Baorong Kang, Zelong Wang, Yuxuan Ma, Xinhua Xia, Hong Yan

**Affiliations:** School of Pharmacy, Hunan University of Chinese Medicine, Changsha 410208, China; 20233789@stu.hnucm.edu.cn (S.L.); 202304030107@stu.hnucm.edu.cn (J.G.); 20243788@stu.hnucm.edu.cn (B.K.); 20243809@stu.hnucm.edu.cn (Z.W.); 202530863@stu.hnucm.edu.cn (Y.M.)

**Keywords:** inhibition of glycolysis, tumor metabolic reprogramming, Warburg effect, targeted therapy, combination therapy

## Abstract

Targeting the Warburg effect (aerobic glycolysis) in tumor cells represents a promising metabolic therapeutic strategy in cancer research. This review analyzes the regulatory mechanisms and therapeutic potential of key glycolysis pathway components, including glucose transporters (GLUTs) and glycolytic enzymes such as hexokinase 2 (HK2), phosphofructokinase (PFK), glyceraldehyde-3-phosphate dehydrogenase (GAPDH), pyruvate kinase M2 (PKM2), and lactate dehydrogenase A (LDHA). We evaluate the molecular mechanisms of various inhibitors and the current clinical development landscape, noting that limitations of monotherapy stem not only from tumor metabolic plasticity but also largely from the unacceptable toxicity of many inhibitors due to the essential role of glycolysis in normal cell metabolism. Furthermore, we explore the molecular basis of synergistic interactions between glycolysis inhibitors and chemotherapy, radiotherapy, immunotherapy, photothermal therapy, and targeted therapy, proposing that rational combination strategies may help overcome resistance and improve therapeutic efficacy. Finally, the review outlines future challenges and directions, emphasizing that the primary obstacle in metabolic treatments is achieving selective inhibition of glycolytic enzymes in cancer cells while sparing normal cells. To address this challenge, the development of high-selectivity agents, cancer-specific nanodelivery systems, precise biomarker identification, and innovative combination regimens based on metabolic-immune regulation is crucial for advancing glycolysis-targeted therapy toward clinical translation.

## 1. Introduction

Cancer remains one of the foremost global public health challenges. According to GLOBOCAN 2022, approximately 20 million new cancer cases and 9.7 million cancer-related deaths were reported worldwide in that year, underscoring the persistent severity of its prevention and treatment landscape [1]. Despite continuous advancements in conventional treatment modalities such as surgery, chemotherapy, and radiotherapy, as well as emerging targeted and immunotherapies, the clinical management of cancer continues to grapple with pivotal challenges, notably recurrence, metastasis, and treatment resistance [2]. The deeper our grasp of cancer biology—particularly the Hallmarks of Cancer—the more promising the development of new therapeutic strategies becomes [3]. Within this context, “metabolic reprogramming” serves as a core feature, unveiling how tumor cells reshape their energy and material metabolic networks to accommodate malignant proliferation. This phenomenon provides new targets for tumor intervention [4,5].

**Figure 1:**
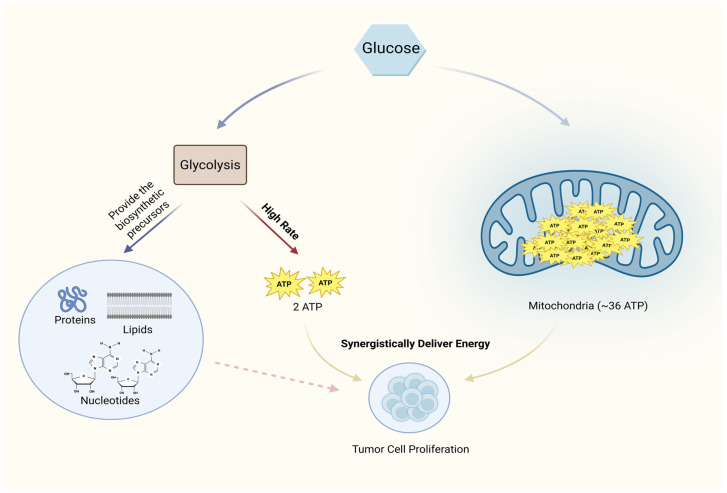
Glycolytic intermediates are shunted into biosynthetic pathways to fuel tumor proliferation (figure was created in https://BioRender.com, accessed on 20 January 2026).

In aerobic conditions, tumor cells preferentially convert glucose to lactate through glycolysis rather than the more efficient mitochondrial oxidative phosphorylation (OXPHOS), a phenomenon known as the Warburg effect or aerobic glycolysis [6]. In tumor cells, glycolytic flux is markedly enhanced compared to normal cells; however, this process does not independently sustain the entire energetic demand of cellular proliferation. Indeed, the net yield of ATP from glycolysis alone—only 2 ATP per molecule of glucose—is significantly lower than the 36 ATP generated via complete OXPHOS. To compensate for this energetic shortfall, tumor cells adopt a coordinated metabolic strategy wherein a portion of the glucose continues to undergo OXPHOS [7,8]. Contemporary cancer metabolism research has clarified that oncogenic signals, notably MYC and HIF-1α, reprogram tumor cell metabolism such that the primary biological significance of elevated glycolytic flux extends beyond mere ATP generation [4,9,10]. While the Warburg effect is often portrayed as an adaptation to hypoxia or a compensatory ATP source, emerging evidence underscores that the anabolic outputs of glycolysis—intermediates that feed the synthesis of nucleic acids, proteins, and lipids—represent a critical, and in many contexts, the principal driver of tumor proliferation [11] (Figure 1). In addition, the final product of glycolysis, lactate, is secreted into the extracellular space, leading to its accumulation in the tumor microenvironment (TME) and causing local acidification [12]. Lactate not only serves as an alternative energy substrate but also functions as a signaling molecule that promotes tumor angiogenesis [13], induces epithelial-mesenchymal transition (EMT) [14], and shapes an immunosuppressive microenvironment [15], hereby comprehensively driving tumor invasion and metastasis [16].

The profound impact of glycolytic metabolites on the TME has made the precise regulatory mechanisms of this pathway a research focus. Glycolysis is composed of a series of enzyme-catalyzed reactions, and its activity is subjected to stringent multi-level regulation. At the transcriptional level, a variety of oncogenes (e.g., MYC) and tumor suppressor genes (e.g., p53) regulate the expression of key glycolysis-related molecules, including glucose transporters (GLUTs) and glycolytic enzymes such as hexokinase (HK), phosphofructokinase (PFK), glyceraldehyde-3-phosphate dehydrogenase (GAPDH), pyruvate kinase (PK), and lactate dehydrogenase (LDH) [17,18,19]. Concurrently, the activity of these pivotal enzymes is also finely tuned by post-translational modifications (PTMs) such as phosphorylation, acetylation, ubiquitination, and the emerging lactylation, enabling dynamic responses to both intracellular and extracellular signals [20,21,22,23].

In addition to these classic regulatory mechanisms, a growing body of evidence suggests that many glycolytic enzymes possess “non-canonical” or “moonlighting” functions, acting as key regulators of gene transcription, apoptosis, and signal transduction, thereby directly influencing tumor progression and therapy response [24,25,26].

Given the pivotal role of glycolysis in maintaining the malignant phenotype of tumors, targeting this pathway has emerged as a promising anti-tumor strategy. Significant progress has been made in the development of inhibitors targeting key nodes within this pathway, with several candidates now advancing into clinical evaluation. However, the intrinsic metabolic heterogeneity of tumors and their robust metabolic plasticity often limit the efficacy of single-target inhibitors and predispose to resistance [27]. Therefore, a thorough dissection of the glycolytic regulatory network, the development of highly selective inhibitors, and the proactive exploration of combination strategies with radiotherapy, chemotherapy, immunotherapy, and other treatment modalities are crucial for enhancing the efficacy of cancer therapy.

This review examines the core mechanisms of tumor glycolysis and its role in the tumor microenvironment, summarizes the development progress and clinical translation status of inhibitors targeting key nodes in this pathway, and explores in depth the synergistic mechanisms of combining glycolytic inhibition with various therapeutic modalities. Finally, this review dissects the challenges and future opportunities in this field, aiming to provide systematic insights and references for promoting the translation of glycolysis-targeted therapies from basic research to clinical practice.

## 2. Metabolic Features of Glycolysis in Tumor Cells and Their Regulatory Mechanisms

The Warburg effect represents one of the most fundamental metabolic hallmarks of tumor cells, and its maintenance is dependent on a complex regulatory network composed of transcription factors, metabolic enzymes, metabolites, and the tumor microenvironment [28,29].

### 2.1. The Molecular Basis of the Warburg Effect

**Figure 2:**
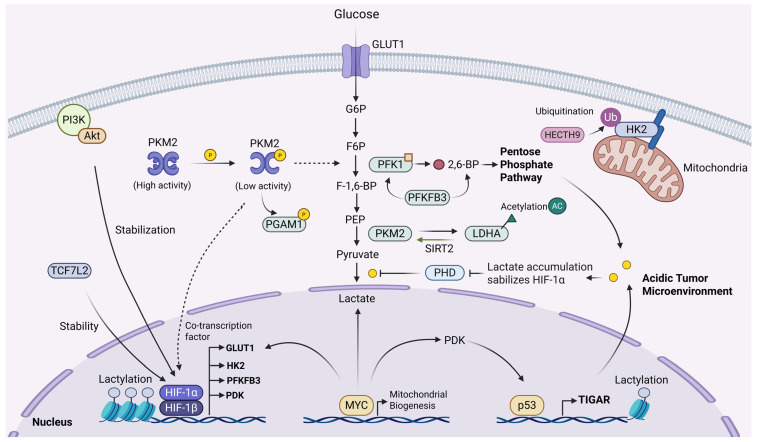
Schematic representation of metabolic reprogramming and the regulation of aerobic glycolysis. Glucose enters the cell via GLUT1 and undergoes glycolysis. PKM2 acts as a central regulator; its phosphorylation converts the high-activity tetramer into a low-activity dimer, shunting metabolic flux toward the Pentose Phosphate Pathway and enabling PKM2 to function as a nuclear co-transcription factor. The pathway terminates with LDHA-mediated lactate production. Accumulated lactate acidifies the microenvironment, stabilizes HIF-1α, and drives nuclear histone lactylation. HIF-1α stability is further supported by PI3K/Akt and TCF7L2 signaling. In the nucleus, the HIF-1α/β complex and MYC upregulate key glycolytic genes (GLUT1, HK2, PFKFB3, PDK), while p53 induces TIGAR to inhibit glycolysis. Collectively, these interactions reinforce the glycolytic shift (figure was created in https://BioRender.com).

The Warburg effect describes the metabolic phenotype in which tumor cells preferentially rely on glycolysis and produce lactate even under normoxic conditions. The maintenance of this phenotype is not determined by a single factor; rather, it is driven by a complex, dynamic regulatory network comprising transcription factors, metabolic enzymes, metabolites, and the TME (Figure 2). A thorough dissection of the multi-level regulatory mechanisms governing this network constitutes the theoretical cornerstone for the development of effective glycolysis inhibition strategies.

The Warburg effect is orchestrated and sustained across multiple layers, including gene transcription, protein functional modifications, and metabolic feedback. At the transcriptional regulation level, a series of key transcription factors act synergistically to systematically reshape the metabolic gene expression profile of tumor cells [30,31,32]. HIF-1α is a core regulator responding to the hypoxic environment within tumors. Upon stabilization, HIF-1α directly activates the transcription of multiple genes encoding glycolytic enzymes, such as HK2, 6-phosphofructo-2-kinase/fructose-2,6-bisphosphatase 3 (PFKFB3), and LDHA, as well as genes involved in glucose uptake, including glucose transporter type 1 (GLUT1), thereby globally enhancing glycolytic flux [33,34,35,36]. Notably, even under normoxic conditions, oncogenic signaling pathways such as PI3K/Akt can maintain a high glycolytic state by stabilizing HIF-1α [37]. For instance, Jin et al. demonstrated that the transcription factor TCF7L2 can regulate the EGLN2/HIF-1α axis, influencing the stability of HIF-1α. Knockdown of TCF7L2 leads to a synchronized downregulation of HIF-1α and its downstream target genes, including the glucose transporter GLUT1 and key glycolytic enzymes such as HK2 and LDHA, confirming the positive regulatory role of the TCF7L2-HIF-1α axis [38]. MYC, a prominent oncogene, collaborates with these factors and plays a broader role in metabolic reprogramming. MYC not only directly activates the transcription of glycolytic enzymes such as GLUT1, HK2, LDHA, pyruvate dehydrogenase kinase (PDK), and PFKFB3, but also promotes mitochondrial biogenesis [39,40,41]. Studies have confirmed that MYC directly activates the transcription of genes encoding GLUT1, HK2, LDHA, PDK, and PFKFB3 [42,43]. Interestingly, research by Austin C et al. found that after MYC overexpression, genes encoding components of the mitochondrial electron transport chain are preferentially induced, while the maximal expression of glycolytic enzyme genes is relatively delayed. This suggests that MYC-driven metabolic reprogramming is a temporally ordered, complex process rather than a simple metabolic switch [44]. In contrast to these pro-glycolytic factors, the tumor suppressor p53 acts as a negative regulator of the Warburg effect. Wild-type p53 can inhibit key nodes of glycolysis (such as fructose-2,6-bisphosphate (F-2,6-BP) levels and phosphofructokinase 1 (PFK1) activity by inducing the expression of genes like TIGAR (TP53-induced glycolysis and apoptosis regulator), thereby promoting OXPHOS. Consequently, frequent mutations or loss of function of p53 in tumors directly relieve the inhibition of glycolysis, facilitating the formation of the Warburg phenotype [29,45,46,47].

Building upon transcriptional regulation, the flux through the glycolytic pathway is also subject to rapid and fine-tuned adjustments by post-translational modifications (PTMs) [48]. Various PTMs modulate the activity, stability, subcellular localization, and protein–protein interactions of key metabolic enzymes, enabling tumor cells to swiftly adapt to microenvironmental changes. PKM2 isoform exemplifies such regulation. Upon growth factor signaling, PKM2 undergoes tyrosine phosphorylation, resulting in the dissociation of its highly active tetramer into a low-activity dimer. This dimeric form of PKM2 can translocate to the nucleus and act as a co-transcription factor interacting with HIF-1α, STAT3, and other factors, directly participating in the regulation of proliferation-related genes, thereby endowing metabolic enzymes with “non-canonical” biological functions [49,50]. For instance, Zhou et al. reported that ULK1-dependent phosphorylation of PKM2 antagonizes its O-GlcNAcylation, thereby regulating the Warburg effect in breast cancer [51]; Wang et al. discovered that PKM2 itself can function as a histidine kinase to phosphorylate phosphoglycerate mutase 1 (PGAM1), enhancing glycolytic flux and tumor growth [52]. In addition, acetylation, ubiquitination, glycosylation, lactylation, and succinylation also widely participate in the regulation of glycolytic enzymes. Acetylation of LDHA can inhibit its activity, while the deacetylase SIRT2 can reverse this process [53]; SIRT2 also deacetylates PKM2 to regulate glycolysis [54]; the E3 ubiquitin ligase HECTH9 mediates K63-linked ubiquitination of HK2, regulating its mitochondrial localization and affecting cellular resistance to apoptosis [55]. O-GlcNAcylation of PFK1 inhibits its activity, redirecting metabolic flux toward the pentose phosphate pathway [56]. Even environmental factors such as smoking can reprogram lung cancer cell metabolism by inducing succinylation of GAPDH [57]. The recently discovered histone lactylation modification, where lactate directly serves as a modification donor, further establishes a direct molecular bridge between metabolic products and epigenetic regulation [53].

Beyond the regulation of protein levels, metabolites within the glycolytic pathway also serve as crucial signaling molecules, providing feedback and feedforward control over metabolic flux through allosteric effects or indirect signaling pathways. While ATP and citrate are classic allosteric inhibitors, AMP, ADP, and F-2,6-BP function as key activators. Among them, F-2,6-BP stands out as the most potent activator, with its concentration being regulated by the bifunctional enzyme PFKFB (such as PFKFB3) [58,59]. In tumors, the overexpression of PFKFB3 leads to elevated levels of F-2,6-BP, which in turn strongly activates PFK1, establishing a positive feedback loop that continuously drives glycolytic flux [60,61]. On the other hand, the end product of glycolysis, lactate, is far from being a mere metabolic waste. The accumulation of intracellular lactate can inhibit the activity of prolyl hydroxylase (PHD), thereby stabilizing HIF-1α. This forms a “lactate-HIF-1α” positive feedback loop, further solidifying and enhancing the Warburg effect as well as the acidic microenvironment of tumors [62]. The aforementioned histone lactylation modification directly exemplifies the signaling function of lactate.

The maintenance of aerobic glycolysis in tumors is an intricately integrated dynamic process. It initiates with a transcriptional network centered around HIF-1α, MYC, and p53, which reprograms the metabolic blueprint in a programmed manner. This is followed by rapid and flexible fine-tuning of enzyme function through various post-translational modifications. Ultimately, the metabolites themselves act as signaling molecules, feeding back into multiple levels of the regulatory network. This complex and precise regulatory system not only explains the robustness of the Warburg effect but also reveals the compensatory and adaptive mechanisms that may be encountered when targeting this pathway. It provides a crucial theoretical perspective for the development of multi-layered and combinatorial targeted therapeutic strategies.

### 2.2. Key Regulatory Targets Associated with Glycolysis

**Figure 3:**
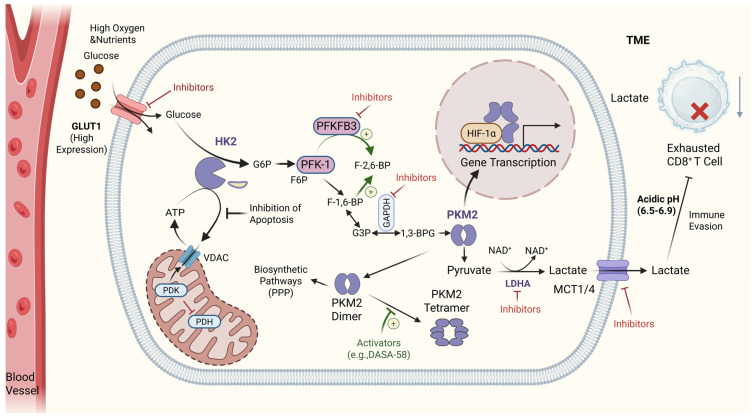
Schematic diagram illustrating key regulatory targets associated with the Warburg effect (figure was created in https://BioRender.com).

The glycolytic comprises a series of enzyme-catalyzed reactions, wherein several pivotal rate-limiting enzymes and transporters frequently exhibit aberrant expression or altered activity within tumor cells. These components have emerged as critical nodes for modulating the Warburg effect and serve as focal points for targeted therapeutic interventions (Figure 3). Targeting these nodes aims to sever the energy supply to tumors, disrupt the provision of biosynthetic precursors, and reverse the immunosuppressive microenvironment sculpted by cancer cells.

#### 2.2.1. GLUTs

GLUTs are not enzymatic components of the glycolytic pathway, they play an essential upstream role in regulating glycolytic flux by controlling glucose availability within tumor cells. Increased expression or membrane localization of specific GLUT isoforms, particularly GLUT1 and GLUT3, has been widely reported across multiple cancer types and is closely associated with enhanced aerobic glycolysis and poor clinical outcomes. By facilitating elevated glucose uptake, GLUTs indirectly sustain the activity of downstream glycolytic enzymes summarized in Table 1, thereby reinforcing the Warburg phenotype. Consequently, GLUTs are commonly classified as glycolysis-associated regulatory targets rather than core glycolytic enzymes, and their inhibition represents an indirect but effective strategy to impair tumor glycolytic metabolism [63]. Among them, GLUT1 is widely overexpressed in various solid tumors, including intrahepatic cholangiocarcinoma, colorectal cancer, and pancreatic cancer. Its expression level is closely associated with tumor aggressiveness, poor prognosis, and therapeutic resistance, involving multiple classic pathways such as PI3K-Akt and HIF-1 [63,64,65]. For instance, in intrahepatic cholangiocarcinoma, high GLUT1 expression is significantly correlated with vascular invasion, advanced TNM stage, and poorer overall survival [66]. In addition to directly promoting tumor cell proliferation, elevated GLUT1 expression also leads to substantial glucose consumption in the TME, causing infiltrating immune cells such as T cells to suffer from “nutrient starvation” and subsequent functional impairment, thereby facilitating immune evasion [67]. Other GLUT family members, such as GLUT3, are highly expressed in tumors like glioblastoma due to their higher glucose affinity, which helps maintain energy supply under low-glucose conditions [68,69]. Large-scale tissue microarray analyses have confirmed that GLUT1 expression in cancers is generally significantly higher than in corresponding normal tissues.

Therefore, inhibiting GLUT1 has emerged as a crucial strategy for reversing tumor metabolism and the immunosuppressive microenvironment. Preclinical studies have demonstrated that anti-GLUT1 antibodies or inhibitors can suppress tumor cell proliferation and enhance sensitivity to chemotherapeutic agents such as cisplatin and paclitaxel [70]. However, therapeutic targeting of GLUTs faces major challenges: firstly, compensatory mechanisms may exist among different GLUT isoforms (e.g., GLUT2, GLUT4), limiting the efficacy of single-target inhibition; secondly, the basal expression of GLUT1 in normal tissues (such as red blood cells and the blood–brain barrier) raises concerns about potential toxicity. Future research needs to further elucidate the isoform-specific expression profiles and compensatory networks across different tumor types, and develop more selective inhibitors or nanodelivery systems to advance clinical translation.

#### 2.2.2. HK2

Following glucose uptake mediated by GLUTs, HK2 catalyzes the first committed enzymatic step of glycolysis by phosphorylating glucose to glucose-6-phosphate (G6P), thereby functionally linking enhanced glucose transport to sustained glycolytic flux. Among the four HK isoforms, HK2 is specifically overexpressed in the vast majority of malignant tumors while its expression is low in normal tissues, making it a highly promising selective anticancer target [71]. For example, in hepatocellular carcinoma (HCC), high HK2 expression is significantly associated with malignant tumor progression and poor patient prognosis [72]. The oncogenic function of HK2 is closely linked to its distinct subcellular localization and functional context in cancer cells. Like HK1, HK2 can bind to the VDAC on the mitochondrial outer membrane [73,74]. In addition to the aforementioned classical metabolic functions, recent studies have also revealed important “non-classical” or “non-metabolic” functions of HK2. For instance, in breast cancer, HK2 has been shown to directly phosphorylate the immune checkpoint protein PD-L1, enhancing its stability and thereby facilitating tumor immune evasion [75]. These multifaceted oncogenic mechanisms make strategies targeting HK2 highly promising. Preclinical studies have demonstrated that knocking down HK2 or applying pharmacological inhibitors can reverse chemoresistance and significantly enhance the sensitivity of tumor cells to traditional chemotherapeutic agents such as cisplatin and paclitaxel [76].

In summary, HK2 plays a central role in tumorigenesis and progression by driving glycolysis, inhibiting apoptosis, and modulating the tumor immune microenvironment. Targeting HK2, especially developing highly selective inhibitors or combining it with chemotherapy and immunotherapy, is a promising direction for future cancer treatment. The current challenge in this field mainly lies in how to maximize targeting tumor cells while minimizing potential toxicity to normal tissues (such as certain blood cells) that express HK2.

#### 2.2.3. PFK

PFK-1 is a major rate-limiting and flux-controlling enzyme in glycolysis, and its activity is primarily regulated by the potent allosteric activator F-2,6-BP [59]. F-2,6-BP is synthesized and degraded by the bifunctional enzymes of the PFKFB family (PFKFB1-4). Among these isoforms, PFKFB3 stands out due to its kinase activity being approximately 700-fold higher than its phosphatase activity, positioning it as a pivotal regulator of intracellular F-2,6-BP levels [77]. Overexpression of the PFKFB3 isoform has been documented across various tumor types. For instance, studies have demonstrated that in renal cell carcinoma (RCC), elevated PFKFB3 expression significantly enhances glycolytic flux and promotes cellular proliferation [78]. This overexpression leads to a sustained increase in intracellular F-2,6-BP levels, which in turn robustly activates PFK-1, thereby markedly augmenting glycolytic throughput [79]. This metabolic reprogramming is not confined to tumor cells alone; PFKFB3 is also highly expressed in tumor-associated endothelial cells [80]. Research indicates that endothelial cell-derived PFKFB3-driven glycolysis is indispensable for angiogenesis. Under hypoxic conditions or upon stimulation by angiogenic factors, PFKFB3 expression in endothelial cells is upregulated. Its knockdown or inhibition impairs endothelial cell proliferation, migration, and tube formation, consequently suppressing both in vivo and in vitro pathological angiogenesis [77]. This suggests that PFKFB3 supports tumor growth and metastasis by facilitating tumor angiogenesis, thereby providing essential nutrients and conduits for tumor progression [81].

Beyond its classical metabolic role, PFKFB3 possesses significant non-metabolic functions. Recent studies have uncovered that PFKFB3 can interact with β-catenin, enhancing its protein stability and thereby modulating the Wnt/β-catenin signaling pathway. For example, in castration-resistant prostate cancer, PFKFB3 promotes tumor cell proliferation by modulating the PI3K/Akt-Wnt/β-catenin axis [82]. Similarly, in anaplastic thyroid carcinoma (ATC), PFKFB3 also facilitates cellular proliferation and migration via the Wnt/β-catenin pathway [83]. These findings reveal a novel mechanism whereby PFKFB3 directly participates in critical signaling pathways to regulate the cell cycle and tumor progression.

In summary, PFKFB3 drives tumor progression across multiple dimensions by integrating metabolic reprogramming, signaling pathway activation, and angiogenesis regulation. Targeting PFKFB3, particularly through the development of highly selective inhibitors and their combination with existing therapies, represents a promising therapeutic strategy. Future research should further explore its role in specific tumor subtypes while addressing challenges related to inhibitor selectivity, potential toxicity, and optimal combination regimens in clinical translation.

#### 2.2.4. GAPDH

GAPDH catalyzes a central step of glycolysis by converting glyceraldehyde-3-phosphate into 1,3-bisphosphoglycerate while reducing NAD^+^ to NADH, thereby directly coupling glycolytic carbon flux to cellular redox homeostasis. Although historically regarded as a housekeeping enzyme, increasing evidence indicates that GAPDH plays a critical role in sustaining the high glycolytic flux characteristic of the Warburg effect in cancer cells [84]. Under conditions of aerobic glycolysis and hypoxia, continuous regeneration of NAD^+^ is required to maintain GAPDH activity, which is predominantly achieved through LDHA-mediated conversion of pyruvate to lactate. This functional coupling between GAPDH and LDHA enables uninterrupted glycolytic throughput and ATP production in highly glycolytic tumor [85].

Importantly, metabolic control analysis has demonstrated that GAPDH can exert substantial control over glycolytic flux specifically in Warburg-phenotype cancer cells, whereas its rate control coefficient is relatively low in normal or less glycolytic cells [11]. Partial inhibition of GAPDH therefore disproportionately suppresses glycolysis in highly glycolytic tumors. Experimental studies further suggest that GAPDH activity must be reduced below a critical threshold (approximately 19–20%) to disrupt glycolytic equilibrium and flux, revealing a potential therapeutic window [11]. Consistent with this concept, pharmacological inhibition of GAPDH using agents such as koningic acid (KA), 3-bromopyruvate (3-BP), or vitamin C (VC) in specific contexts selectively impairs glycolysis, induces energetic stress, and suppresses tumor growth in glycolysis-dependent cancers [86,87]. Beyond its classical metabolic function, GAPDH also exhibits diverse non-glycolytic (“moonlighting”) roles that contribute to tumor progression, survival, and therapy response. These functions are regulated by post-translational modifications, including S-nitrosylation, oxidation, and acetylation, as well as by subcellular localization [88]. Under oxidative or nitrosative stress, GAPDH can translocate to the nucleus, where it participates in the regulation of apoptosis, autophagy, and gene transcription. However, cancer cells frequently suppress this pro-apoptotic pathway; for example, Akt-mediated phosphorylation or mutant p53-dependent cytoplasmic retention of GAPDH favors sustained glycolysis and tumor cell survival [89].

In addition, GAPDH has been implicated in a range of non-metabolic processes, including vesicle trafficking, maintenance of DNA integrity, tRNA export, epithelial–mesenchymal transition, and resistance to anticancer therapies [88,90]. In certain tumor types, such as neuroblastoma, GAPDH supports metabolic adaptation, cell survival, and potentially nutrient transport, further underscoring its multifaceted oncogenic roles [91]. Conversely, aberrant aggregation or mislocalization of GAPDH can contribute to cell death under specific therapeutic conditions, highlighting the context-dependent nature of its functions.

In summary, GAPDH serves as a central integrator of glycolytic flux, redox balance, and non-canonical signaling pathways in cancer. Through its dual roles in sustaining the Warburg effect and modulating tumor cell fate decisions, GAPDH promotes tumor growth, survival, and therapeutic resistance, positioning it as a promising antiglycolytic target in highly glycolysis-dependent cancers.

#### 2.2.5. PKM2

PKM2 is the final rate-limiting enzyme in the glycolytic pathway, catalyzing the conversion of PEP into pyruvate and ATP [92]. Unlike the highly active tetrameric PKM1 found in most normal tissues, tumor cells predominantly express PKM2 in a low-activity dimeric form [93]. This form of PKM2 exhibits a slower catalytic rate, leading to the accumulation of upstream glycolytic intermediates (such as 3-phosphoglycerate), which are then diverted into biosynthetic pathways like the PPP. This metabolic reprogramming provides tumor cells with abundant precursors for nucleic acids, lipids, and amino acids, facilitating rapid proliferation [94]. The “enzyme activity switch” characteristic of PKM2 makes it a core component of metabolic reprogramming.

Beyond its role as a metabolic enzyme, the dimeric PKM2 can translocate to the nucleus under stimulation by growth factors (such as epidermal growth factor), where it functions as a protein kinase or a transcriptional co-activator [95]. In the nucleus, PKM2 can phosphorylate histone H3 or interact with transcription factors such as HIF-1α and STAT3, thereby regulating the expression of downstream target genes and promoting tumor growth [96]. The expression and activity of PKM2 are regulated by complex mechanisms, including m6A RNA methylation and circRNA/miRNA networks [97]. Its aberrant overexpression is closely associated with poor prognosis and the formation of an immunosuppressive microenvironment in various cancers (such as lung cancer, colorectal cancer, and liver cancer) [98]. The tumor-promoting mechanisms of PKM2 are not limited to tumor cells alone. For instance, in myeloid-derived suppressor cells (MDSCs), PKM2-dependent glycolysis is activated, enhancing their immunosuppressive functions and thereby promoting colorectal cancer development [99]. In Cancer-Associated Fibroblasts (CAFs) the nuclear translocation of PKM2 can lead to sustained activation of the NF-κB signaling pathway, further maintaining the pro-inflammatory and pro-fibrotic state of the tumor microenvironment [100]. Given the core role of PKM2, strategies targeting its different functional states (such as promoting tetramer formation, inhibiting nuclear translocation, or blocking protein interactions) are being explored. For example, the small molecule activator DASA-58 can induce the formation of highly active PKM2 tetramers, inhibit its nuclear translocation and HIF-1α signaling, thereby suppressing pathological angiogenesis [101].

In summary, PKM2 drives tumor metabolic reprogramming, proliferation, angiogenesis, and immune evasion through its unique “enzyme activity switch,” nuclear translocation-mediated gene regulation, and complex expression regulatory networks. This makes it a highly promising multi-dimensional anticancer target.

#### 2.2.6. LDHA

LDHA, a pivotal enzyme catalyzing the conversion of pyruvate to lactate, represents the terminal step of the Warburg effect and sustains glycolytic flux by regenerating NAD^+^. In tumors, LDHA is markedly upregulated, leading to excessive lactate production and secretion. This accumulation of lactate profoundly remodels the TME and drives malignant progression [102,103]. Tumor cells employ multiple mechanisms to elevate LDHA expression. For instance, the transcription factor FOXQ1 directly activates LDHA transcription, while histone modifications, such as H3K18 lactylation, form positive feedback loops with c-Myc and HIF-1α, further amplifying LDHA expression and lactate generation. Post-translational modifications of LDHA, such as K118 acetylation, also enhance its enzymatic activity and stability [104]. While extracellular acidification is a multifactorial process involving additional contributors beyond lactate extrusion, LDHA-driven lactate production remains a dominant and therapeutically actionable mechanism shaping the acidic and immunosuppressive tumor microenvironment [102,105]. For instance, mathematical modeling studies have suggested that CO_2_ produced by tumor cell respiration can diffuse and accumulate within poorly perfused TME, where it is catalyzed by carbonic anhydrases such as CAIX to form carbonic acid—representing an additional pivotal mechanism underlying extracellular hypercarbia and acidification [106]. Moreover, the highly active proton extrusion systems in tumor cells, including V-ATPase and the Na^+^/H^+^ exchanger NHE1, also directly contribute to the extracellular H^+^ concentration [107,108]. This multifactorial acidic microenvironment not only directly promotes tumor cell invasion, metastasis, and angiogenesis but also facilitates extracellular matrix remodeling, thereby creating conditions conducive to tumor dissemination. Moreover, the acidic environment and high lactate concentrations directly impair the metabolism and function of CD8^+^ T cells and Natural Killer (NK) cells. Lactate inhibits the glycolytic capacity of CD8^+^ T cells, interferes with IFN-γ signaling, and weakens their proliferation and cytotoxic activity [109].

Consequently, LDHA serves as a critical metabolic enzyme that maintains the high glycolytic rate of tumors and, through the generation of an acidic, immunosuppressive TME, drives tumor immune evasion. Targeting LDHA and its downstream lactate signaling provides a dual strategy for improving cancer prognosis through metabolic intervention combined with immunotherapy.

#### 2.2.7. Other Potential Targets and Combination Strategies

A comprehensive glycolysis inhibition network must also consider other critical nodes that maintain metabolic homeostasis. Monocarboxylate transporters (MCTs, particularly MCT1 and MCT4) are responsible for exporting lactate from the cell, which is crucial for maintaining intracellular pH and exacerbating extracellular acidity [110,111,112]. Their inhibitors, such as the clinically investigated AZD3965, which has so far not demonstrated objective tumor responses in phase I/II trials, with only isolated cases of stable disease and a single confirmed complete response, indicating limited clinical benefit [113,114]. Because lactate shuttling (the process of lactate transfer between cells, also known as the “lactate shuttle”) between hypoxic (export) and oxygenated (import/oxidize) compartments is frequently mediated by MCT4 and MCT1, respectively, selective MCT1 inhibition may be insufficient in MCT4-high tumors and motivates dual-transporter strategies or rational combinations. PDK locks metabolic flux into glycolysis by phosphorylating and inhibiting PDH. Agents targeting PDK, such as dichloroacetate (DCA), inhibit PDK activity, thereby activating PDH and forcing tumor cells to shift their metabolism from glycolysis towards OXPHOS [115,116,117]. However, the mechanism of action of metformin is distinct. It primarily limits OXPHOS capacity by inhibiting mitochondrial electron transport chain complex I, leading to reduced ATP production and a compensatory increase in glycolysis [118,119,120]. Therefore, in preclinical models, the combined administration of DCA and metformin generates a dual metabolic inhibition: DCA inhibits glycolytic flux, while metformin inhibits oxidative phosphorylative metabolism. This synergistic effect induces severe metabolic stress and energy depletion in tumor cells, thereby sensitizing them to anticancer therapies [121].

Importantly, glycolytic reprogramming is shaped not only by core metabolic enzymes but also by lineage- and context-specific regulators. Enolase 2 (ENO2), also known as neuron-specific enolase, exemplifies such a regulator by linking glycolysis to tumor lineage identity, hypoxia adaptation, and malignant progression. ENO2 supports aerobic glycolysis under hypoxic conditions and promotes tumor cell proliferation, survival, migration, invasion, and epithelial–mesenchymal transition. In head and neck squamous cell carcinoma, ENO2 coordinates PKM2-dependent glycolytic and non-glycolytic pathways [122], whereas in other tumors, such as clear cell renal cell carcinoma, it is frequently upregulated downstream of HIF-1α to sustain energy metabolism and tumor progression [123,124]. Moreover, ENO2 has been associated with chemoresistance and poor prognosis across multiple cancer types, underscoring its role as a lineage-associated metabolic regulator that complements core glycolytic nodes [125].

Despite these advances, clinical translation of glycolysis-targeted therapies remains challenging due to metabolic plasticity, compensatory pathway activation, and potential toxicity to normal tissues, including lactate shuttle immune cells. Future strategies should therefore emphasize tumor-selective targeting, biomarker-guided patient stratification, and rational combination regimens that simultaneously disrupt multiple metabolic nodes—such as PFKFB3, MCTs, and lineage-specific regulators like ENO2—while integrating metabolic interventions with immunotherapy and signal transduction inhibitors to achieve durable therapeutic responses.

### 2.3. Regulation of Glycolytic Metabolism by the TME

**Figure 4:**
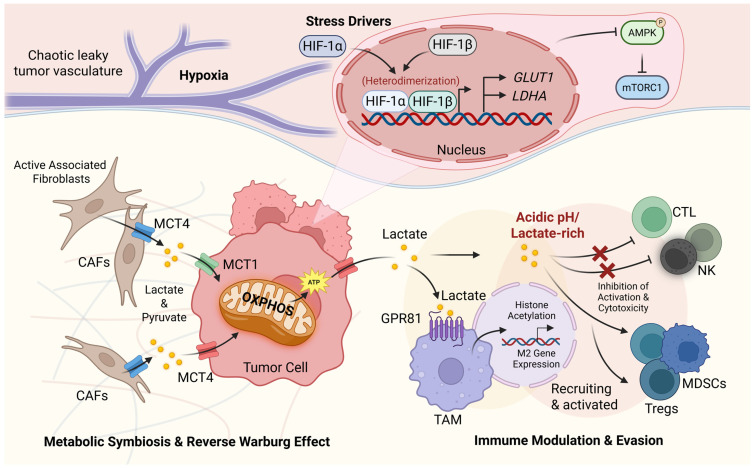
Metabolic Crosstalk and Immunosuppression within the TME (figure was created in https://BioRender.com).

Tumor cells do not exist in isolation; instead, they coexist with surrounding stromal cells, immune cells, vasculature, and the extracellular matrix, collectively forming a complex TME. The diverse components within the TME engage in intricate metabolic crosstalk, jointly regulating tumor glycolysis, thereby influencing tumor progression, immune evasion, and therapeutic responses (Figure 4).

A quintessential model of metabolic crosstalk is the Reverse Warburg Effect, which is a key manifestation of the broader “lactate shuttle” operating within the tumor microenvironment [12,126]. In this model, CAFs induced by tumor cells, undergo aerobic glycolysis. Critically, this process typically occurs in CAFs that are situated near blood vessels and are therefore relatively well-oxygenated, allowing them to retain oxidative capacity. These CAFs produce and secrete substantial amounts of lactate and pyruvate into the microenvironment [127,128]. Adjacent tumor cells, which are often in hypoxic regions, then capture these metabolites via Monocarboxylate Transporters (MCTs, such as MCT1 and MCT4) and channel them into the mitochondria for OXPHOS, efficiently generating ATP and supporting rapid tumor proliferation [128,129]. This spatial and metabolic relationship defines two functional classes of tumor cells: (1) Oxidative tumor cells located near vessels, which rely on OXPHOS and can utilize lactate as an energy source (lactate-consuming cells) [130,131,132]; (2) Glycolytic tumor cells residing in hypoxic areas, where very hypoxic cells (pO_2_ under 0.5%) may lose the ability to switch to OXPHOS. This metabolic compartmentalization is supported by the presence of CAFs, which can act as lactate producers through aerobic glycolysis and further sustain the oxidative tumor cell population [133,134,135]. This metabolic heterogeneity and symbiosis, underpinned by lactate shuttling, confer significant flexibility and adaptability for tumor survival and development [136]. Similar metabolic coupling exists between tumor cells and other stromal components; for instance, in pancreatic cancer, CAFs supply lactate to tumor cells, which preferentially utilize glutamine [137].

The glycolytic metabolism within the TME profoundly reshapes the immune compartment. Accumulated lactate acts not merely as a metabolic waste product but as a pivotal immunoregulatory molecule. High concentrations of lactate can promote the polarization of Tumor-Associated Macrophages (TAMs) towards an M2 phenotype through signaling via the G Protein-Coupled Receptor 81 (GPR81) and by fueling the tricarboxylic acid (TCA) cycle to drive histone acetylation and M2-related gene expression [138,139,140]. However, lactate alone is not sufficient for this transformation. Cytokines such as IL-4, IL-13, IL-10, TGF-β, and CSF-1 can achieve M2 polarization even in the absence of lactate, underscoring the critical role of cytokine signaling in the metabolic crosstalk between TME cells and cancer cells [62,141,142]. Concurrently, the lactate-rich and acidic pH environment exerts strong inhibitory effects on effector immune cells. It suppresses the activation, proliferation, and cytotoxic functions of Cytotoxic T Lymphocytes (CTLs) and NK cells. Moreover, the lactate milieu facilitates the recruitment and activation of immunosuppressive cells such as Regulatory T cells (Tregs) and MDSCs, further exacerbating immune evasion [143].

Hypoxia, acidosis, and nutrient deprivation—prevalent stress conditions within the TME—are intrinsic drivers of glycolytic metabolism. Hypoxia stabilizes HIF-1α, directly upregulating the transcription of numerous glycolytic genes, including GLUTs and LDHA, thereby enhancing the glycolytic capacity of tumor cells. Additionally, the energy stress sensor AMP-Activated Protein Kinase (AMPK) becomes activated under these conditions, helping cells adapt to metabolic stress and maintain survival by inhibiting pathways such as mTORC1. AMPK activity is closely linked to tumor metabolic reprogramming and immune responses [144,145]. The metabolic heterogeneity among different cell types within the TME, as well as within tumor cells themselves, provides tumors with remarkable adaptability. For instance, while some tumor cells primarily engage in glycolysis, others rely on oxidative metabolism. This heterogeneity enables tumors to cope with dynamic changes in oxygen and nutrient availability within the TME [146]. The unique metabolic states of CAFs, endothelial cells, and immune cells, through the exchange of metabolites such as lactate, glutamine, and fatty acids, collectively construct a metabolic network that supports tumor growth and metastasis.

In summary, the TME precisely regulates glycolysis through multi-layered mechanisms, including metabolic symbiosis, immune metabolic reprogramming, and stress signal activation. Targeting metabolic key nodes in the TME, such as inhibiting monocarboxylate transporters (MCTs), modulating the metabolic state of CAFs, or reversing lactate-mediated immunosuppression, has emerged as a promising anticancer strategy. Future research should aim to further elucidate the spatiotemporal dynamics and molecular details of metabolic crosstalk within the TME, thereby developing more effective combination therapies to improve the prognosis of cancer patients.

## 3. Classification of Tumor Glycolytic Metabolism-Related Inhibitors

Based on a deep understanding of the key nodes in the glycolysis pathway, researchers have developed a variety of targeted inhibitors with the aim of inhibiting tumor growth by blocking the energy supply and biosynthesis of tumor cells. These inhibitors can be classified into the following categories according to their targets (Table 2).

### 3.1. Inhibitors of Glucose Uptake (GLUTs Inhibitors)

GLUT1 serves as the primary portal for glucose uptake in tumor cells. Its expression is significantly upregulated under the influence of key oncogenic signaling pathways such as HIF-1α and PI3K/AKT/mTOR, establishing it as a core molecule that sustains the Warburg effect and drives tumor malignancy. Consequently, the development of GLUT1 inhibitors to sever tumor energy supply at its source has emerged as a focal point in cancer metabolic therapy. Currently, small-molecule GLUT1 inhibitors are primarily categorized into three generations. First-generation inhibitors (e.g., STF-31) demonstrated the feasibility of targeting GLUT1, selectively inhibiting glucose uptake in VHL-deficient renal cancer cells and inducing necrotic death [147,148]. However, these early inhibitors exhibit limited potency and selectivity, resulting in insufficient monotherapy efficacy and highlighting the challenges of early-stage inhibitors in translational medicine. The advent of second-generation high-selectivity inhibitors marks a significant breakthrough in this field. Among them, BAY-876 stands out as one of the most potent known GLUT1 inhibitors. Despite its impressive in vitro potency (IC_50_ 2 nM) and robust anti-tumor activity across various preclinical models, including ovarian cancer and triple-negative breast cancer, it has not progressed to clinical trials. Its mode of action extends beyond merely blocking glucose uptake; it induces profound metabolic reprogramming, leading to reactive oxygen species (ROS) accumulation and mitochondrial dysfunction, ultimately triggering apoptosis. Studies have shown that BAY-876 can synergize with chemotherapeutic agents or other targeted drugs (such as T2R agonists), offering promising avenues for combination therapy [149,150,151,152,153]. The authors suggest that the primary reasons for the lack of clinical translation include the compound’s classification as a chemical probe rather than a drug candidate, concerns regarding systemic toxicity due to GLUT1’s basal expression in normal tissues (e.g., blood–brain barrier, erythrocytes), and insufficient IND (Investigational New Drug) application data. Another representative molecule, WZB117, exerts its effects through competitive binding to the substrate site of GLUT1. These findings are primarily derived from preclinical in vitro and in vivo studies, which represent a lower level of evidence compared with clinical trials. Recent research has uncovered its broader anti-tumor mechanisms: in melanoma, WZB117 enhances the efficacy of apatinib by blocking the STAT3/PKM2 signaling axis [154]; in breast and colon cancers, it effectively reverses resistance to radiotherapy and chemotherapy, indicating its significant potential in overcoming therapeutic resistance [155,156]. However, similar to BAY-876, WZB117 has also faced challenges in clinical development, primarily due to limited data on its pharmacokinetics, potential off-target effects, and a lack of robust clinical trial evidence.

Despite the active research on small-molecule inhibitors, emerging modalities such as monoclonal antibodies [157] and gene silencing therapies based on nanotechnology (e.g., siRNA) [158] are gaining attention for their potential to reduce off-target toxicity through higher targeting specificity. For instance, specific antibodies against GLUT1 have demonstrated anti-tumor efficacy in preclinical models. However, the clinical translation of GLUT1 inhibitors still faces formidable challenges. Firstly, the basal expression of GLUT1 in normal tissues such as the blood–brain barrier and erythrocytes raises concerns about systemic inhibition potentially leading to neurotoxicity and anemia [149,159]. Secondly, the high metabolic plasticity and redundancy of tumor metabolism mean that a single blockade of glucose uptake may be compensated by alternative metabolic pathways, resulting in suboptimal monotherapy outcomes. This underscores the necessity of combination therapy [63]. Future strategies may involve combination with immune checkpoint inhibitors to modulate the immunosuppressive microenvironment or synergistic blockade of other metabolic nodes (such as hexokinase or lactate dehydrogenase). Additionally, developing biomarkers (such as GLUT1 protein expression levels, specific tumor subtypes, or genetic mutation profiles) to predict patient response is crucial for achieving precision medicine.

In summary, research on GLUT1 inhibitors has transitioned from proof-of-concept to the development of high-selectivity drugs and exploration of combination strategies. Despite challenges related to toxicity and resistance mechanisms, rational design of combination therapies, utilization of nanodelivery systems to enhance tumor targeting, and patient stratification based on biomarkers hold promise for GLUT1-targeted approaches to become innovative metabolic interventions in cancer treatment. Future research should focus on elucidating resistance mechanisms and conducting rigorous translational studies in these areas.

### 3.2. Inhibitors of Key Glycolytic Enzymes

Based on the canonical glycolytic pathway outlined in Table 1, several enzymatic steps function as key regulatory nodes and have therefore attracted considerable interest as therapeutic targets in cancer. In particular, enzymes catalyzing irreversible or rate-limiting reactions—such as hexokinase, phosphofructokinase-1, and pyruvate kinase—play critical roles in controlling glycolytic flux and metabolic reprogramming in tumor cells. Consequently, pharmacological inhibition of these enzymes, as well as modulation of their upstream regulators, has emerged as a promising strategy to disrupt tumor glycolytic metabolism. The major classes of glycolysis-associated inhibitors and their mechanisms of action are discussed below. It should be noted that, while Table 1 summarizes the core enzymatic steps of glycolysis, several agents discussed in the following sections exert their antitumor effects through broader metabolic interference rather than strict single-enzyme inhibition.

#### 3.2.1. HK Inhibitors

HK2, as the first rate-limiting enzyme in glycolysis, is a pivotal target for tumor metabolic reprogramming. HK2 inhibitors primarily exert their effects through two mechanisms: (1) directly inhibiting its catalytic activity, and (2) disrupting its association with mitochondria, thereby severing the energy supply to tumor cells and inducing cell death. 2-Deoxy-D-glucose (2-DG), a glucose analog, is one of the earliest and most extensively investigated competitive inhibitors of hexokinase. In addition to its classical mechanism of inhibiting glycolytic flux through competition with glucose, 2-DG has been reported in preclinical studies to induce cellular energy stress, increase ROS production, and disrupt N-linked glycosylation, leading to endoplasmic reticulum stress and apoptosis in tumor cells [160]. Despite these mechanistic insights, the therapeutic efficacy of 2-DG as a single agent has been limited. Clinical evaluation of 2-DG in oncology has largely been restricted to early-phase trials, primarily exploring its feasibility and tolerability in combination with radiotherapy or chemotherapy. For example, a phase I study combining 2-DG with docetaxel in patients with advanced prostate cancer mainly assessed safety and showed limited antitumor activity [161], while a phase I/II trial in glioma patients combined 2-DG with radiotherapy but yielded modest clinical outcomes [162]. To date, no late-phase clinical trials have demonstrated clear therapeutic benefit, and further clinical development of 2-DG in cancer therapy appears to be limited.

3-BP is a highly reactive alkylating agent originally characterized as an inhibitor of HK2, capable of disrupting its mitochondrial association and thereby impairing glycolytic flux. However, accumulating evidence indicates that the antitumor activity of 3-BP is not restricted to HK2 inhibition alone [163,164]. Proteomic and biochemical analyses have demonstrated that 3-BP interacts with a broad spectrum of cellular proteins involved in glycolysis, mitochondrial metabolism, redox regulation, and stress response pathways, reflecting its pleiotropic and non-selective mode of action. This extensive protein reactivity contributes to profound metabolic collapse and cell death, but also underlies its significant systemic toxicity, which has severely limited its clinical translation [165]. To mitigate these limitations, recent studies have explored nanotechnology-based delivery strategies aimed at improving tumor selectivity and reducing off-target effects. Nanocarriers such as metal–organic frameworks and bismuth sulfide-based nanosystems have been reported to enhance intratumoral accumulation and mitochondrial targeting of 3-BP, thereby improving therapeutic efficacy while partially alleviating systemic toxicity [166]. These approaches have also shown potential synergistic effects when combined with radiotherapy or immunotherapy in preclinical models [167].

Lonidamine is another glycolysis-associated inhibitor with a well-documented but multifaceted mechanism of action. Although initially described as an inhibitor of HK2 through disruption of its interaction with the VDAC on the outer mitochondrial membrane, subsequent studies have revealed that Lonidamine exerts broader metabolic effects [168,169]. In addition to suppressing glycolysis, Lonidamine interferes with mitochondrial oxidative phosphorylation, leading to impaired ATP production, altered mitochondrial membrane potential, and increased susceptibility to apoptosis [168,170,171]. Recent research has further focused on elucidating the downstream signaling pathways associated with Lonidamine-induced metabolic stress and on developing novel derivatives or nano-formulations to improve its solubility, bioavailability, and tumor selectivity. These strategies aim to enhance therapeutic efficacy while overcoming resistance mechanisms and minimizing adverse effects [172,173].

In summary, classic HK2 inhibitors are limited by issues of selectivity or toxicity, and the development of highly selective HK2 inhibitors remains a current research focus. The research on HK2 inhibitors has evolved from early broad-spectrum metabolic inhibition to a precise intervention phase that emphasizes targeting, synergy, and new mechanisms of action (such as protein degradation). These advancements lay a solid foundation for the development of the next generation of highly effective and low-toxicity antitumor metabolic therapies.

#### 3.2.2. PFKFB3 Inhibitors

PFKFB3 inhibitors attenuate glycolytic flux by reducing the allosteric activator F-2,6-BP levels, thereby indirectly inhibiting PFK1 activity. PFK158 (ACT-PFK158) stands as the pioneering PFKFB3 inhibitor to enter clinical trials. Preclinical investigations have demonstrated its capacity to suppress tumor cell proliferation and angiogenesis, as well as to target cancer stem cells by downregulating markers such as CD133 and SOX2, ultimately inhibiting tumor sphere formation and enhancing chemosensitivity [174]. In gynecological tumor models, PFK158 has been shown to induce lipophagy and cell death [175]. However, its Phase I clinical trial (NCT02044861) outcomes highlighted the challenges of monotherapy. Early reports indicated good tolerance at low doses and noted a case of reduced tumor burden in a patient with liver metastasis [176]. Nevertheless, due to limited efficacy, the trial was ultimately terminated. This result prompted researchers to reconsider the viability of using PFKFB3 inhibitors as standalone treatments for advanced solid tumors, given the potential bottlenecks posed by metabolic pathway redundancy and compensatory mechanisms.

In contrast to early inhibitors, AZ67 is a small molecule inhibitor with high selectivity for PFKFB3, validated through isothermal titration calorimetry [177]. Intriguingly, studies have revealed that AZ67’s anti-angiogenic effects are independent of its glycolysis inhibition [178]. In models such as glioblastoma, AZ67 effectively suppresses tumor cell invasion and migration. Its mechanism may involve disrupting the metabolic coupling between tumor cells and the microenvironment, for instance, by reducing lactate production and interfering with the lactate-driven HIF-1α stabilization feedback loop that promotes invasion [179]. Consequently, PFKFB3 inhibitors may exert broader antitumor effects by modulating the tumor microenvironment. However, despite AZ67’s impressive performance in preclinical studies, it has yet to advance into clinical trials. Its development has been primarily hindered by limitations in its pharmacokinetic properties; in mouse studies, a relatively high dose administered via intravenous injection was required to achieve sufficient concentrations in blood and brain tissue, suggesting that its oral bioavailability and systemic exposure may be inadequate [180]. Additionally, early in vivo toxicological assessments have revealed potential safety concerns, indicating a narrow therapeutic window. To overcome efficacy bottlenecks, new-generation inhibitors continue to emerge. Among them, KAN0438757 not only inhibits glycolysis but also impairs DNA homologous recombination repair, leading to the accumulation of DNA damage within tumor cells and producing a “synthetic lethality” effect. This significantly enhances radiosensitivity [181], providing a robust theoretical basis for the combined application of PFKFB3 inhibitors with radiotherapy or DNA-damaging chemotherapeutic agents.

The setbacks encountered in the PFK158 clinical trial indicate that future PFKFB3 inhibitor development must shift towards more precise strategies. Inhibiting a single metabolic target is prone to compensation, making combination therapies a promising avenue. PFKFB3 inhibitors have the potential to normalize aberrant tumor vasculature, improve drug delivery, and synergize with agents such as bevacizumab [182,183]. Given the high expression of PFKFB3 in regulatory T cells and other immune cells, its inhibitors may modulate the immune microenvironment, and combining them with PD-1/PD-L1 inhibitors could potentially overcome immune resistance [184]. Building on the mechanism of KAN0438757, such combination strategies can directly attack the fundamental survival mechanisms of tumor cells, holding significant translational potential [185]. Moreover, there is a need to identify biomarkers that can predict therapeutic efficacy, such as the expression ratio of PFKFB3-specific splice variants, particular gene mutations, or PET-CT imaging characteristics, to achieve patient stratification and precision therapy. To address the issue of poor water solubility observed in some inhibitors, employing delivery systems like nanoliposomes or polymeric micelles can enhance tumor targeting and reduce systemic toxicity, representing a crucial technical pathway for improving efficacy [60,186].

In summary, as a pivotal node in tumor metabolism, the development of PFKFB3 inhibitors has evolved from early broad-spectrum glycolysis inhibition to a current stage characterized by high selectivity and novel mechanisms of action, such as disrupting DNA repair. Although monotherapy efficacy faces challenges, through rational combination therapy strategies, biomarker-guided patient selection, and advanced drug delivery technologies, targeting PFKFB3 remains poised to become a significant component in the future landscape of cancer treatment. Future research should focus on deepening the understanding of resistance mechanisms and conducting rigorous translational studies in the aforementioned directions.

#### 3.2.3. GAPDH Inhibitors

Regulatory strategies targeting GAPDH primarily rely on enzymatic inhibition, reflecting its central position as a flux-controlling node in glycolysis and its essential role in maintaining NAD^+^/NADH redox balance [187,188]. Inhibition of GAPDH disrupts ATP production, biosynthetic precursor supply, and redox homeostasis simultaneously, rendering highly glycolytic tumor cells particularly susceptible to energetic collapse [189,190]. KA, a fungal-derived natural product, is a highly potent and selective irreversible inhibitor of GAPDH. Its mechanism involves covalent modification of the catalytic cysteine residue (Cys152) in the active site, leading to efficient enzyme inactivation [191,192]. This selectivity underpins its ability to preferentially kill highly glycolytic cancer cells while sparing normal cells, as validated in models of thyroid cancer, where KA suppressed GAPDH activity, cell proliferation, and migration [193]. Xu et al. discovered that KA targets GAPDH, inhibiting glycolysis and suppressing the proliferation of neuroendocrine prostate cancer (NEPC) cells. KA induces mitochondrial dysfunction, elevates ROS, and activates apoptosis by downregulating phospho-Akt and GSK-3β signaling. In vivo studies demonstrate that KA significantly reduces tumor growth and proliferation, highlighting its therapeutic potential for NEPC [194].

More recently, the development of DC-5163, a novel small-molecule GAPDH inhibitor, has provided important translational validation of GAPDH as a therapeutically actionable metabolic target. Unlike classical inhibitors, DC-5163 directly suppresses GAPDH activity with improved selectivity, leading to inhibition of aerobic glycolysis, depletion of intracellular ATP pools, and disruption of redox balance in cancer cells [195]. Preclinical studies demonstrated that DC-5163 significantly reduces glucose uptake, lactate production, and tumor growth in vivo, while exhibiting a more favorable tolerability profile compared with earlier GAPDH-targeting compounds [196]. Compared to classic GAPDH inhibitors such as KA, DC-5163 may exhibit more moderate inhibitory activity, but its advantages lie in higher selectivity and potentially superior safety [197]. Early GAPDH inhibitors were often limited in clinical translation due to lack of selectivity or significant toxicity. In contrast, DC-5163 demonstrates favorable tolerability toward normal cells while effectively inhibiting tumors, endowing it with more advantageous pharmacological characteristics [198]. These findings establish DC-5163 as a mechanistically validated, next-generation GAPDH inhibitor that serves as a crucial bridge between foundational biochemical experiments and the development of clinically deployable metabolic therapies.

Collectively, these studies demonstrate that direct or indirect inhibition of GAPDH can selectively compromise tumor cell viability and synergize with blockade of complementary metabolic pathways. However, clinical translation remains challenging due to limited selectivity and toxicity, underscoring the need for more precise GAPDH-targeting strategies, predictive biomarkers, and rational combination regimens.

#### 3.2.4. PKM2 Modulators

The regulatory strategies targeting PKM2 are primarily categorized into inhibitors and activators, accurately reflecting its “dual functionality” in tumors as both a metabolic enzyme and a protein kinase or transcriptional co-activator [199].

PKM2 inhibitors aim to suppress its pro-tumor activities. The classic representative, the natural product Shikonin, specifically binds to and stabilizes the low-activity dimeric form of PKM2, thereby blocking glycolytic flux, leading to an energy crisis and inducing strong oxidative stress and apoptosis [200,201,202]. In addition to Shikonin, novel synthetic inhibitors are constantly emerging. For instance, the naphthoquinone derivative C3k exhibits nanomolar-level inhibitory activity against PKM2, effectively suppressing the proliferation of tumor cells with high PKM2 expression and inducing autophagic cell death [203]. Another study identified the clinical drug Benserazide through virtual screening, which directly binds and inhibits PKM2, demonstrating superior anti-tumor effects compared to Shikonin in melanoma models and overcoming BRAF inhibitor resistance [204].

In contrast, PKM2 activators such as TEPP-46 (ML-265) promote the formation of the high-activity tetrameric PKM2 by binding to the subunit interface, thereby accelerating the final step of glycolysis [205,206]. This seemingly paradoxical “acceleration” strategy actually depletes upstream glycolytic intermediates used for biosynthesis (e.g., nucleotides, amino acids), thus inhibiting tumor cell proliferation—a phenomenon validated in various models, including lung cancer [207]. More importantly, activating PKM2 can remodel the metabolic phenotype of tumor cells, shifting them from a high reliance on glycolysis to a greater dependence on mitochondrial OXPHOS. This shift renders them more sensitive to OXPHOS inhibitors (e.g., Metformin) or glucose analogs (e.g., 2-DG), providing a theoretical basis for synergistic combination therapies [208].

Despite the promising preclinical research, the clinical translation of PKM2 modulators still faces challenges. For example, Shikonin’s limited selectivity and toxicity issues restrict its clinical application [209]. The efficacy of activators like TEPP-46 in maintaining PKM2 tetramerization within the complex in vivo environment also requires further optimization. Future directions include developing compounds with higher selectivity and better pharmacokinetic properties, exploring biomarker-based patient stratification strategies, and designing rational combination therapy regimens (e.g., combined with immune checkpoint inhibitors) to overcome tumor metabolic redundancy and achieve clinical breakthroughs.

#### 3.2.5. LDHA Inhibitors

LDHA catalyzes the conversion of pyruvate to lactate, and its overexpression is a hallmark of the Warburg effect in tumors. Inhibition of LDHA not only directly weakens tumor cell glycolytic energy supply by reducing ATP generation and NAD^+^ regeneration, but also lowers lactate concentrations within the TME. This reversal of lactate-mediated suppression of cytotoxic T cells and natural killer cells facilitates the reprogramming of anti-tumor immunity [210,211]. Consequently, LDHA serves as an ideal target that bridges tumor metabolism and immunotherapy. GSK2837808A, a highly selective LDHA inhibitor, has been shown in preclinical studies to rapidly suppress lactate production across various cancer cell lines [212,213,214]. Notably, treatment of melanoma cells with GSK2837808A significantly enhances the cytotoxic activity of T cells [215], providing direct evidence for its potential in combination immunotherapy.

FX11, a reversible competitive LDHA inhibitor, exhibits anti-tumor activity across multiple preclinical models [216,217,218]. Recent studies have demonstrated that FX11 selectively impairs glycolysis in AML cells, inducing cell death while sparing healthy hematopoietic progenitors, thereby underscoring its therapeutic promise [219]. In solid tumor models such as neuroblastoma, FX11 also inhibits oxidative glycolysis and induces cell cycle arrest [220]. Compared to earlier compounds, ML-05 exhibits stronger potency and distinct immunomodulatory functions. Studies demonstrate that local injection of ML-05 not only suppresses B16F10 melanoma growth and reduces intratumoral lactate levels but also activates Th1 and GMZB^+^ CD8^+^ T cell subsets within the tumor. When combined with anti-PD-1 antibodies or STING agonists, it produces synergistic antitumor effects, highlighting its potential as an immune sensitizer.

**Table 2:** Representative Inhibitors Targeting Glycolysis and Glucose Transport: Mechanisms of Aerobic Glycolysis Inhibition.

The Inhibitory Target	Representative Drugs	Mechanisms of Glycolysis Inhibition	References
GLUT1	BAY-876	High-Efficiency and Selective Inhibition of Glucose Transport	[221]
GLUT1	WZB117	Competitive inhibition of net glucose uptake in tumor cells, leading to an energy crisis	[222]
HK2	2-DG	Competitive inhibition of HK2 reduces the phosphorylation of normal glucose	[223]
HK2	Lonidamine	Inhibition of HK2 significantly reduces glycolytic flux in tumor cells	[224]
HK2	3-BP	Inhibition of key glycolytic enzymes such as HK2 directly blocks glucose catabolism and ATP production	[225]
PFKFB3	PFK158	Inhibition of F-2,6-BP generation reduces glucose uptake, ATP generation, and lactate release in tumor cells	[226]
PFKFB3	AZ67	Direct binding and inhibition of PFKFB3 leads to a decrease in intracellular F-2,6-BP, thereby suppressing the activity of PFK-1 and reducing lactate production and ATP levels	[227]
GAPDH	KA	Irreversibly inhibits GAPDH by covalently modifying its catalytic cysteine, thereby blocking the GAPDH step in glycolysis and leading to ATP depletion	[228,229]
GAPDH	DC-5163	Diminishes glucose uptake and lactate production in cancer cells, thereby restricting energy supply and inducing apoptosis	[195]
PKM2	Shikonin (Inhibitors)	Inhibition of dimeric PKM2 kinase activity significantly reduces glucose uptake and lactate production levels	[230]
PKM2	Benserazide (Inhibitors)	Direct binding and blockade of PKM2 enzymatic activity suppress aerobic glycolysis while upregulating OXPHOS	[204]
PKM2	TEPP-46 (Activator)	Promoting PKM2 tetramer formation prevents its nuclear translocation and inhibits the activity of transcription factors such as HIF-1α; tetrameric PKM2 reduces the biosynthetic precursors required for tumor cell growth, thereby interfering with anabolic metabolism	[208,231]
LDHA	GSK2837808A	Selective inhibition of LDHA catalytic activity significantly reduces lactate production in cancer cells, leading to a decrease in extracellular acidification rate (ECAR) and thus suppressing the glycolytic process	[213,232]
LDHA	FX11	Inhibition of LDHA activity disrupts tumor glycolytic metabolism, induces the production of ROS, and leads to ATP depletion	[233,234]
LDHA	ML-05	Inhibition of tumor lactate production and cell proliferation reduces ATP and ROS generation, thereby inducing cell cycle arrest	[210,235]
MCT1	AZD3965	Inhibition of lactate transmembrane transport interferes with tumor energy metabolism	[236]

Despite encouraging preclinical results, the clinical translation of LDHA inhibitors faces challenges. Metabolic pathway redundancy and compensatory mechanisms may limit monotherapy efficacy. Future development should focus on the following aspects: combination with immune checkpoint inhibitors (e.g., anti-PD-1/PD-L1) to simultaneously dismantle metabolic and immune suppression barriers, exploring synergistic combinations with DNA-damaging chemotherapeutic agents, developing next-generation inhibitors with higher selectivity and improved pharmacokinetics, and exploring prodrug strategies and nano-delivery systems to enhance tumor targeting and reduce systemic toxicity.

In summary, LDHA inhibitors provide a dual-faceted strategy against tumors by targeting the core of tumor metabolism, offering both energy deprivation and immune reversal pathways. Although no LDHA inhibitor has yet been successfully marketed, novel inhibitors like ML-05, which demonstrate significant potential in activating antitumor immunity, lay a solid theoretical foundation for their combination with immunotherapy. Overcoming current challenges and advancing rational combination regimens into clinical evaluation remain central to future developments in this field.

### 3.3. Clinical Development of Glycolysis Inhibitors and Reasons for Failure

Targeting the heightened glycolytic flux in cancer cells, known as the Warburg effect, has been a long-standing therapeutic strategy. Several small-molecule inhibitors of glycolysis have progressed into clinical trials, but with limited success. Table 3 reviews key candidates and analyzes the factors contributing to their clinical failures.

Targeting glycolysis is theoretically an attractive strategy; however, its clinical translation faces multiple challenges, including metabolic network redundancy, drug delivery difficulties, and safety concerns. Currently, the trend leans towards using these inhibitors as “tumor metabolic sensitizers,” combining them with chemotherapy, radiotherapy, or immunotherapy to overcome resistance and enhance therapeutic efficacy.

## 4. Glycolysis Inhibitors in Cancer Treatment: Combination Therapeutic Strategies

**Figure 5:**
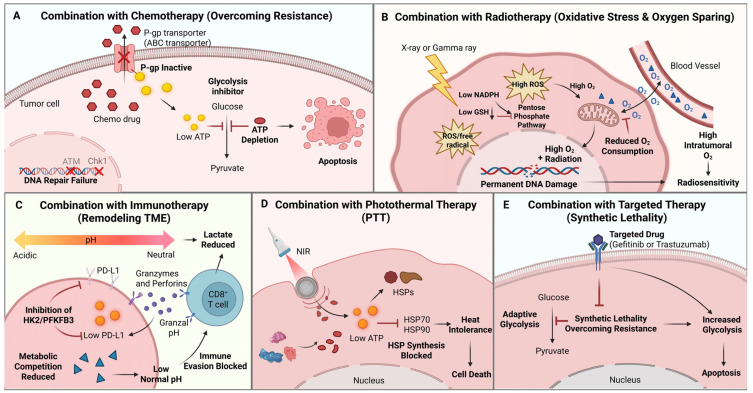
Multimodal Synergistic Mechanisms of Glycolysis Inhibitors in Cancer Therapy. (**A**) Chemotherapy: Glycolysis inhibition causes ATP depletion, inactivating P-gp efflux pumps and impairing DNA repair mehanisms. (**B**) Radiotherapy: Disruption of redox homeostasis (decreased NADPH/GSH) amplifies ROS damage, while reduced oxyen consumption alleviates hypoxia (Oxygen Sparing). (**C**) Immunotherapy: Reduction in lactate secretion normalizes TME pH, reactivating effector T cells and downregulatng PD-L1 expression. (**D**) Photothermal Therapy (PTT): Energy deprivation prevents the synthesis of Heat Shock proteins (HSPs), sensitizing cells to hyperthermia. (**E**) Targeted Therapy: Blocking gycolysis cuts off the metabolic adaptive pathway used by resistant cells (e.g., EGFR-mutant), inducing synthetic lethality (figure was created in https://BioRender.com).

Given the complexity and plasticity of tumor metabolism, the clinical efficacy of glycolysis inhibitors as monotherapy is limited. Consequently, current research focuses on combining these agents with conventional therapies (chemotherapy, radiotherapy) and emerging modalities (immunotherapy, targeted therapy) to achieve synergistic effects (Figure 5).

### 4.1. Combined Application with Chemotherapy

The combination of glycolysis inhibitors and chemotherapeutic agents has a solid theoretical foundation. The synergistic mechanisms extend beyond simple energy deprivation, encompassing multi-level regulation of cellular stress and death pathways to overcome metabolic plasticity and chemoresistance in tumors [242,243]. Key Synergistic Mechanisms: (1) Induction of Energy Crisis and Apoptosis Sensitization: Inhibiting key glycolytic enzymes (e.g., HK2, PFKFB3) or glucose transporters (e.g., GLUT1) rapidly depletes ATP in tumor cells [244]. This energy crisis undermines the cells’ ability to perform energy-intensive processes such as DNA damage repair, ion pump maintenance, and resistance to intrinsic apoptosis signals, thereby lowering the threshold for DNA damage-induced apoptosis and mitochondrial apoptosis [245]. (2) Reversal of Chemotherapy Resistance: Multi-drug resistance (MDR), often linked to overexpression of ATP-binding cassette (ABC) transporters (e.g., P-gp/ABCB1), is a major cause of chemotherapy failure. Glycolysis inhibitors limit the supply of ATP, effectively inhibiting the function of these efflux pumps, increasing intracellular drug concentrations, and reversing resistance phenotypes [87]. Additionally, glycolytic metabolic flux provides essential substrates for post-translational modifications (e.g., acetylation, lactylation) of DNA repair proteins. Therefore, glycolysis inhibition directly interferes with key pathways such as homologous recombination repair (HR) [246,247,248,249]. Multiple studies have confirmed that 2-DG or PFKFB3 inhibitors (e.g., 3PO) can sensitize cells to platinum-based drugs or PARP inhibitors by reducing ATP levels, with validation in ovarian and pancreatic cancer models [250,251]. (3) Improvement of TME and Drug Delivery: Glycolysis inhibition significantly reduces lactate secretion, alleviating TME acidosis [252]. This not only stabilizes and enhances the activity of pH-sensitive chemotherapeutic agents (e.g., anthracyclines) but also improves tumor vasculature abnormal contraction caused by acidosis, enhancing blood perfusion and drug penetration for better intratumoral delivery [253]. An improved TME also helps mitigate chemotherapy-induced immunosuppression [254,255]. (4) Synthetic Lethality and Overcoming Metabolic Compensation: For tumors with specific metabolic dependencies, combination therapy can induce synthetic lethality. For example, in osteosarcoma, the combination of glycolysis inhibitors with low-dose methotrexate shows significant anti-tumor effects [256]. To overcome metabolic compensation where tumor cells switch to OXPHOS following glycolysis inhibition, the “dual-pathway energy blockade” strategy has emerged. For instance, the natural product Gnetin H not only inhibits glycolysis but also synergizes with mitochondrial respiratory chain inhibitors (e.g., metformin). When combined with chemotherapy, it achieves a more comprehensive blockade of tumor energy supply, providing a new avenue to overcome resistance [257].

Beyond the classic 2-DG, the combination of the PFKFB3 inhibitor (PFK158) with carboplatin in preclinical studies can significantly reverse ovarian cancer chemoresistance by inducing autophagic cell death [258]. Moreover, the combination of LDHA inhibitors with gemcitabine exhibits a powerful synergistic effect in pancreatic ductal adenocarcinoma (PDAC) models [259]. In the field of glioblastoma (GBM), the combination strategy of targeting PKM2 with temozolomide (TMZ) effectively inhibits aerobic glycolysis and enhances chemosensitivity [260].

In terms of clinical translation, several clinical trials are actively exploring this approach. For instance, Phase I dose-escalation trial assesses the safety and preliminary efficacy of 2-DG alone or in combination with docetaxel for the treatment of advanced solid tumors [261,262]. These studies provide a crucial bridge for the translation of glycolysis inhibitors from the laboratory to the clinic.

In summary, although this combination strategy holds great promise, it still faces several challenges. These include the potential toxicity of glycolysis inhibitors to normal tissues (especially the brain and heart), the metabolic heterogeneity within tumors leading to variability in therapeutic efficacy, and the lack of reliable biomarkers for predicting response. Future research directions should focus on developing more tumor-selective inhibitors (such as nanotechnology-based targeted delivery systems), exploring the optimal sequencing and dosing of combination therapies, and utilizing metabolic imaging (such as novel PET tracers) to monitor therapeutic responses in real-time and guide personalized treatment.

### 4.2. Combined Application with Radiotherapy

The efficacy of radiotherapy (RT) is severely limited by tumor cell metabolic reprogramming, particularly the Warburg effect leading to enhanced glycolysis. This metabolic state not only exacerbates tumor hypoxia by rapidly depleting glucose and oxygen but also produces abundant lactate and antioxidant precursors (e.g., NADPH) that help tumor cells resist RT-induced oxidative and DNA damage [263]. Consequently, targeting glycolysis has emerged as a promising strategy to enhance radiosensitivity, primarily operating across three interrelated levels.

First, glycolytic inhibitors directly amplify RT cytotoxicity by disrupting tumor oxidative homeostasis. RT primarily kills cells through the generation of ROS. High glycolytic flux fuels the PPP to generate NADPH (maintaining reduced glutathione to scavenge ROS), while the end product lactate can stabilize transcription factors such as Nrf2, upregulating antioxidant genes [264]. Inhibiting key glycolytic enzymes, such as HK2 or LDHA, can simultaneously reduce NADPH supply and lactate accumulation, leading to a dramatic decline in intracellular ROS clearance capacity. For example, the HK2 inhibitor 3-BP effectively depletes intracellular glutathione, rendering tumor cells extremely sensitive to RT-induced oxidative damage [265,266]. Recent studies further reveal that this exacerbated oxidative stress can trigger ferroptosis and other novel forms of programmed cell death, synergizing with RT for enhanced anti-tumor effects [167].

Second, glycolytic inhibitors reverse metabolic abnormality-driven RT resistance signaling pathways. RT itself can induce the stabilization and activation of HIF-1α, subsequently upregulating genes like GLUT1 and VEGF, promoting glycolysis, angiogenesis, and cell survival, thus forming a positive feedback loop that drives RT resistance. Glycolytic inhibitors can break this loop at multiple points. On one hand, direct inhibition of glycolysis reduces lactate production, thereby relieving lactate-mediated inhibition of PHD and promoting the oxygen-dependent degradation of HIF-1α [267]. On the other hand, many glycolytic enzymes (e.g., PFKFB3, PKM2) possess non-metabolic functions, directly participating in DNA damage repair or regulating pro-survival signals such as Akt and c-Myc. Inhibiting these enzymes not only cuts off energy and biosynthetic supply but also directly weakens tumor cells’ damage repair capacity and proliferative signals. Studies have shown that PFKFB3 inhibitors effectively suppress homologous recombination repair in glioma and head and neck cancer models, significantly enhancing RT efficacy [268]. Clinical data also confirm that high PFKFB3 expression correlates with adverse responses to adjuvant RT in breast cancer patients [269].

Third, glycolytic inhibitors indirectly augment RT sensitivity by improving the TME hypoxia. The high oxygen consumption rate of tumor cells, often referred to as the “oxygen hijacking” effect, is a major cause of chronic hypoxia within the TME. Inhibiting glycolysis forces tumor cells to reduce their oxygen consumption rate, temporarily increasing intratumoral oxygen partial pressure and improving hypoxic regions. This “oxygen-sparing” effect has been validated by various inhibitors. For instance, pretreatment with LDHA inhibitors or the glucose analog 2-DG significantly enhances tumor oxygenation in multiple solid tumor models, sensitizing previously RT-resistant hypoxic cells [270]. Improving hypoxia not only directly enhances the oxygen-dependent killing effect of RT but also reverses hypoxia-mediated immunosuppression, laying the groundwork for combined immunotherapy [271].

Despite robust preclinical evidence, the clinical translation of glycolytic inhibitors faces challenges such as systemic toxicity, tumor metabolic heterogeneity, and compensatory mechanisms. Currently, clinical trials exploring the safety of 2-DG combined with RT are ongoing [272]. Future strategies should focus on: (1) developing tumor-targeted delivery systems (e.g., nanoparticles) to improve selectivity—nanomaterials loaded with 3-BP have shown promising RT-sensitizing effects [273]; (2) exploring triple or quadruple combinations with immune checkpoint inhibitors and DNA damage repair-targeted drugs; (3) utilizing biomarkers (e.g., PET-CT-detected glycolytic activity) to select patient cohorts most likely to benefit.

### 4.3. Combination with Immunotherapy

The efficacy of tumor immunotherapy is often limited by the immunosuppressive TME. Recent studies have found that targeting the glycolysis pathway, which tumor cells heavily rely on, can remodel the TME from multiple dimensions, making it a promising strategy to enhance the efficacy of immune checkpoint inhibitors (ICIs). The synergy primarily stems from the ability of glycolysis inhibitors to systematically correct metabolic and immune dysregulation in the TME. 

Firstly, glycolysis inhibitors can reverse the acidosis and metabolic competition in the TME, directly releasing suppressed effector immune cells. The large amount of lactate produced by tumor glycolysis lowers the pH of the TME, which directly damages the metabolic function, proliferative capacity, and cytokine production of CTLs, while promoting the aggregation and activation of Tregs and MDSCs [274,275,276]. Inhibiting LDHA can significantly reduce lactate levels, restore physiological pH in the TME, and thus revive the antitumor activity of CTLs and NK cells [277,278,279]. Preclinical studies have confirmed that the LDHA inhibitor can effectively enhance the therapeutic response to PD-1 antibodies in melanoma models [280]. In addition, the high glucose uptake by tumor cells leads to “nutrient deprivation” for effector T cells, and glycolysis inhibitors can alleviate this metabolic competition, creating conditions for the restoration of T cell function.

Secondly, key enzymes in glycolysis have been found to directly regulate the expression of immune checkpoint molecules, providing molecular targets for combination therapy. HK2, in addition to its classic metabolic function, can act as a protein kinase to phosphorylate IκBα, activating the NF-κB pathway, thereby directly upregulating the transcription of PD-L1 [75]. In gastric cancer, mesenchymal stem cell-derived IL-8 promotes HK2 phosphorylation and nuclear translocation via the CXCR2/AKT axis, which, together with HIF-1α, drives PD-L1 expression [281]. Similarly, PFKFB3 can also promote PD-L1 expression by stabilizing HIF-1α [282]. Therefore, inhibiting these glycolytic enzymes can not only cut off the tumor’s energy supply but also directly downregulate PD-L1, overcoming or preventing primary or secondary resistance to ICIs.

More importantly, the improved TME is conducive to the activation of antigen-presenting cells, thereby initiating and maintaining persistent antitumor immunity. Acidic environments inhibit the maturation and antigen-presenting function of dendritic cells (DCs). LDHA-deficient tumor cells can promote the cross-sensitization of DCs and enhance the activation and tumor infiltration of tumor antigen-specific CD8+ T cells [235]. This indicates that glycolysis inhibition not only lifts immunosuppression but also positively enhances the immune initiation stage, forming a virtuous cycle that may induce long-term immune memory.

Activated T cells are glycolysis-dependent for rapid proliferation and effector functions, yet tumor-targeted glycolysis inhibition can be realized without impairing T cell immunity via multiple mechanisms [244]. Tumor cells specifically overexpress glycolytic enzyme isoforms PKM2 and HK2, while resting and memory T cells predominantly use PKM1 and HK1; thus, selective inhibition of PKM2 or HK2 starves tumor cells but spares T cells [231,283]. T cells exhibit metabolic flexibility to switch to OXPHOS and FAO pathways under glycolysis suppression, whereas tumor cells with mitochondrial defects lack such reprogramming ability and are more vulnerable to glycolytic blockade [284]. Additionally, nanoparticle-mediated targeted delivery and intermittent dosing enable spatial-temporal selective inhibition, which concentrates drugs in tumors and allows T cell metabolic recovery. Preclinical evidence further confirms that glycolysis inhibitors combined with ICIs exert synergistic antitumor effects, offsetting the theoretical risk of T cell inhibition. Collectively, these mechanisms verify the feasibility of targeting tumor glycolysis to preserve even enhance the function of tumor-infiltrating T cells.

Based on the solid mechanistic research mentioned above, the combination therapy of glycolysis inhibitors and ICIs has entered the clinical translation stage. Clinical trials are currently evaluating the safety and efficacy of combining PFKFB3 and PKM2 inhibitors with PD-1/PD-L1 antibodies (e.g., NCT02044861, NCT07197151). In addition, new inhibitors targeting LDHA are also being explored in combination with CTLA-4 antibodies, aiming to achieve breakthroughs in various solid tumors such as melanoma and lung cancer [285,286]. These clinical explorations signify that metabolic immunotherapy is moving from theory to practice, promising new avenues for cancer treatment.

### 4.4. Synergistic Application with Photothermal Therapy (PTT)

PTT, owing to its spatiotemporal controllability and minimally invasive nature, has emerged as a promising strategy for localized tumor treatment. However, its clinical efficacy is often limited by the inherent thermoresistance of tumor cells and the immunosuppressive TME. In recent years, targeting the abnormally active aerobic glycolysis in tumors has emerged as a disruptive strategy for enhancing the efficacy of PTT. By cutting off the energy lifeline of tumor cells, glycolysis inhibitors can overcome PTT barriers from multiple angles, thereby achieving synergistic therapeutic enhancement [287].

The key defense mechanism by which energy-deprivation sensitizes PTT tumor cells to cope with heat stress is the rapid upregulation of heat shock proteins (HSPs, such as HSP70 and HSP90) to repair heat-damaged proteins. The synthesis and function of HSPs are highly ATP-dependent. Glycolysis inhibitors target key rate-limiting enzymes such as HK and PKM2, significantly reducing intracellular ATP levels in tumor cells, thereby fundamentally suppressing HSP synthesis and weakening heat protection capability [288]. For instance, lonidamine, a HK2 inhibitor, encapsulated in liposomes for co-delivery with the photothermal agent IR780, effectively downregulates HSP expression by inhibiting glycolysis and reducing ATP, thereby significantly enhancing the efficacy of PTT in hepatocellular carcinoma [289]. Similarly, the “metabolic starvation-photothermal” therapy based on 2-DG has been widely validated to reverse heat tolerance in various tumor models [290]. The latest nano-strategies aim for dual or multiple metabolic inhibition, such as nanoplatforms co-loading the glycolysis inhibitor metformin and glucose oxidase. These systems intensify ATP depletion through dual pathways of HK2 inhibition and glucose deprivation, dramatically enhancing PTT sensitization [291].

The aberrant metabolism of tumors creates a hypoxic and acidic immunosuppressive TME, which not only hinders PTT but also limits the efficacy of other treatments such as photodynamic therapy (PDT). Glycolysis inhibitors alleviate TME acidosis by reducing lactate production [292]. More importantly, inhibition of glycolysis can decrease the oxygen consumption rate of tumor cells, thereby improving intratumoral hypoxia. Alleviation of hypoxia not only enhances the efficacy of PTT itself—since hyperthermia-induced damage is more effective in an oxygen-rich environment—but also creates favorable conditions for combined PDT, as PDT relies heavily on oxygen to generate cytotoxic ROS [293]. Additionally, the reduction in lactate levels indirectly inhibits the stabilization of HIF-1α, thereby disrupting the positive feedback loop of “glycolysis–lactate–HIF-1α–glycolytic enzyme transcription” that promotes tumor progression [290].

To achieve precise co-delivery of glycolysis inhibitors and photothermal agents and maximize their synergistic effects, multifunctional nanoplatforms have been extensively developed. These systems not only integrate therapeutic components but also often incorporate targeting ligands, imaging agents, and stimulus-responsive release mechanisms. Liquid metal nanoparticles are designed to simultaneously inhibit glycolysis and mitochondrial metabolism, thereby enhancing PTT through comprehensive energy deprivation while exhibiting good biocompatibility [287]. Gold nanostar–branched polymer systems have been employed to deliver the hexokinase inhibitor 3-BP, achieving specific targeting of breast cancer stem cells and facilitating combined metabolic-PTT therapy [294]. Polydopamine (PDA) coatings are widely utilized due to their excellent photothermal conversion efficiency and loading capabilities. For instance, Fe_3_O_4_@PDA nanoparticles loaded with the glycolysis inhibitor Bufalin can target the SRC-3/HIF-1α pathway to inhibit glycolysis and downregulate HSPs, thereby significantly enhancing the efficacy of mild PTT against colorectal cancer [295].

Despite the broad prospects in this field, it continues to face numerous challenges. Future research should prioritize the development of more intelligent and biodegradable nanocarrier systems to effectively address the issue of long-term toxicity. Additionally, a deep exploration of tumor metabolic heterogeneity is crucial for identifying glycolysis inhibition targets that are either broadly applicable or individualized. Simultaneously, there is a need to further elucidate the molecular mechanisms linking metabolism, immunity, and hyperthermia, particularly their specific roles in cancer stem cells and metastatic lesions. Finally, the advancement of meticulously designed clinical trials to validate the safety and efficacy of this combined strategy in humans remains essential

### 4.5. Combination with Targeted Therapy

Targeted therapeutic agents, such as EGFR-TKIs, BRAF inhibitors, and ALK inhibitors, demonstrate significant efficacy in the early stages of treatment. However, tumor cells often develop adaptive resistance through dynamic metabolic reprogramming, which has become a primary cause of treatment failure [296]. Among these adaptive mechanisms, enhanced aerobic glycolysis serves as a crucial strategy for resistant cells to maintain energy supply, biosynthesis, and redox homeostasis [297]. Consequently, targeting the glycolytic pathway not only directly attacks the metabolic vulnerabilities of resistant cells but also synergizes with existing targeted drugs, potentially inducing synthetic lethality. This approach offers a highly promising strategy for overcoming resistance.

Multiple targeted therapy-resistant cells exhibit marked activation of the glycolytic pathway. For example, in EGFR-mutant non-small-cell lung cancer (NSCLC), cells resistant to gefitinib display high expression of the glucose transporter GLUT1, resulting in increased glucose uptake. Co-administration of a GLUT1 inhibitor (e.g., WZB117) with gefitinib effectively reverses resistance and induces apoptosis in these cells [298,299]. Similarly, in BRAF V600E-mutant melanoma, resistance to vemurafenib is closely associated with up-regulation of PFKFB3; combined use of a PFKFB3 inhibitor significantly suppresses the growth of resistant cells and enhances the therapeutic efficacy of vemurafenib [300,301]. Collectively, these studies demonstrate that targeting key glycolytic enzymes can effectively cut off the “metabolic escape route” employed by tumor cells under targeted therapy pressure.

In addition to glycolysis, tumor cells often exhibit a compensatory shift towards OXPHOS when glycolysis is inhibited. This metabolic plasticity is mediated by a network of signaling pathways. For instance, overexpression of UGCG (UDP-glucose ceramide glucosyltransferase) in breast cancer cells induces a metabolic shift characterized by the simultaneous upregulation of both glycolysis and OXPHOS, driven by alterations in sphingolipid composition and enhanced mitochondrial turnover [302,303]. Moreover, in endocrine therapy–tolerant persister cells of ER+ breast cancer, a marked reliance on mitochondrial respiration has been identified as a survival mechanism, indicating that OXPHOS can serve as an alternative energy source under therapeutic pressure [303].

Preclinical studies have shown that simultaneous targeting of glycolysis and OXPHOS can overcome metabolic compensation and induce synergistic anti-tumor effects. For example, the combination of 2-DG, a glycolysis inhibitor, with metformin, an OXPHOS inhibitor, has demonstrated synergistic inhibition of tumor growth, migration, and invasion across multiple cancer types (e.g., ovarian, pancreatic, and breast cancers) through the activation of stress pathways such as p38 MAPK/JNK and AMPK [304,305,306]. Additionally, dual targeting of mitochondrial and glycolytic pathways using metformin and 2-DG has been shown to effectively inhibit proliferation in both highly glycolytic and poorly glycolytic pancreatic cancer phenotypes, suggesting a broad applicability of this combination strategy [305,307,308]. While preclinical data support the efficacy of targeting glycolysis and OXPHOS, the clinical translation of such strategies is not without challenges. The metabolic plasticity of cancer cells can lead to adaptive resistance; for instance, tumors may upregulate alternative fuel sources, such as fatty acid oxidation, in response to glycolytic inhibition, thereby diminishing the long-term efficacy of the treatment [309]. Additionally, the systemic inhibition of these fundamental metabolic pathways may affect normal proliferating tissues, leading to potential toxicities such as lactic acidosis or neurotoxicity [310,311]. Therefore, careful dose optimization and patient selection are critical for balancing therapeutic benefits with safety concerns.

The combined inhibition of glycolysis and specific signaling pathways can induce a synthetic lethal effect, delivering a double blow to tumor cells. A typical example is observed in HER2-positive breast cancer, where HK2 not only regulates glycolysis but also upregulates PD-L1 expression by stabilizing its mRNA, thereby mediating immune evasion. The combination of HK2 inhibitors with trastuzumab, while suppressing tumor metabolism, can downregulate PD-L1 and enhance antibody-dependent cellular cytotoxicity (ADCC), thus overcoming resistance [312]. Additionally, CAFs secrete factors such as CTHRC1 to drive tumor cell glycolysis, forming a positive feedback loop that promotes EGFR-TKI resistance; targeting this loop (e.g., using GA) can effectively reverse resistance [296]. These findings reveal a deep interaction between metabolism and signaling networks, providing new targets for designing combination strategies.

Despite substantial preclinical evidence, the clinical application of glycolysis inhibitors in combination with targeted therapies still faces significant challenges. A major concern is the systemic toxicity associated with glycolytic inhibition. Clinical studies have reported that 2-DG can cause hypoglycemia-like symptoms, including flushing, sweating, and drowsiness, as well as severe lactic acidosis, especially when combined with other metabolic stressors [313]. Furthermore, the non-selective nature of many glycolytic inhibitors raises concerns about off-target effects on normal tissues that rely heavily on glucose metabolism, such as the brain and heart [240,314]. These toxicities may limit the feasible dosing of glycolytic inhibitors in patients, potentially reducing the therapeutic window for achieving synthetic lethality when combined with targeted agents [315]. Therefore, future strategies must prioritize the development of tumor-selective metabolic inhibitors or optimized dosing regimens to mitigate these adverse effects.

Developing reliable biomarkers is crucial, representing a core direction for future research. Utilizing ^18^F-FDG PET/CT functional imaging to dynamically monitor tumor glycolytic activity, or detecting metabolic gene mutations through liquid biopsies, can aid in selecting patients most likely to benefit from combined therapy. Given that glycolysis serves as a vital energy source for normal cells, especially the brain, heart, and blood cells, next-generation inhibitors need to enhance selectivity for tumor cells or manage toxicity through adjusted dosing schedules. Inhibiting glycolysis may force tumor cells to shift toward alternative metabolic pathways (e.g., OXPHOS, glutamine metabolism). Therefore, future exploration should focus on triple therapy regimens—combining glycolysis inhibition, targeted therapy, and a third metabolic inhibitor—to prevent compensatory resistance. Lastly, there is an urgent need for rigorously designed early-phase clinical trials, emphasizing pharmacokinetic/pharmacodynamic relationships, optimal combination regimens, and predictive efficacy biomarkers. Incorporating metabolic inhibitors into the treatment sequence after targeted therapy resistance is a pivotal step toward achieving personalized precision therapy [316,317].

## 5. Conclusions and Outlook

Targeting tumor glycolytic metabolism represents a highly promising new strategy for overcoming resistance to conventional cancer therapies. This review illustrates that key nodes including GLUT1, HK2, PFKFB3, GAPDH, PKM2, and LDHA exert distinct yet complementary functions in driving the Warburg effect and shaping an immunosuppressive tumor microenvironment. However, preclinical and early clinical evidence indicates that inhibition of a single metabolic node can be readily circumvented via adaptive metabolic reprogramming. Accordingly, future therapeutic development should prioritize strategic innovation rather than merely identifying additional inhibitors with similar mechanisms of action.

Of note, the Warburg effect should not be regarded as an all-or-nothing shutdown of mitochondrial respiration. Instead, aerobic glycolysis and OXPHOS typically coexist in tumors, and their relative contributions form a tunable continuum modulated by oxygen availability, redox balance, oncogenic signaling, and microenvironmental constraints. Therefore, effective therapeutic targeting of tumor glycolytic metabolism will likely require three key components: (1) precise biomarker-driven stratification of tumor metabolic states; (2) tumor-selective delivery systems to minimize toxicity toward normal tissues; and (3) rational combination regimens that restrict compensatory metabolic rewiring, including lactate shuttling and mitochondrial adaptation.

Beyond these well-recognized factors, emerging evidence highlights that systemic physiological and demographic variables also act as critical regulators of tumor metabolism. Circadian rhythms govern the rhythmic expression of glycolytic enzymes and transporters in a time-dependent manner, implying that chronotherapeutic strategies may influence treatment efficacy [318]. The gut microbiome generates metabolites such as short-chain fatty acids, which modulate immune cell function and indirectly reshape the metabolic demands of the tumor microenvironment [319]. Furthermore, sex-based differences have been documented: estrogen signaling in female patients frequently promotes oxidative metabolism, whereas androgen pathways in male patients may favor enhanced glycolytic flux [320,321]. Collectively, these factors introduce an additional layer of complexity to the metabolic continuum and should be integrated into the design of therapeutic regimens.

To translate these insights into clinical practice, several strategic shifts are required. First, drug design must evolve toward greater precision and specificity. Next-generation inhibitors should display enhanced tumor selectivity to alleviate metabolic disturbances in normal tissues. The application of nanodelivery systems for targeted accumulation and controlled release at tumor sites represents a critical approach to improve the therapeutic index and reduce off-target toxicity. Second, combination therapies must be mechanistically rationalized rather than relying on empirical drug stacking. Successful combinations should be grounded in a thorough understanding of the crosstalk between metabolic, signaling, and immune networks. For example, combining glycolytic inhibitors with immune checkpoint blockade can simultaneously relieve the “metabolic constraints” and “immune suppression” imposed by tumors, thereby achieving synergistic anti-tumor effects. Similarly, strategies involving dual blockade of metabolic pathways—such as targeting compensatory OXPHOS or glutamine metabolism activated in resistant tumors—may effectively prevent or reverse therapeutic resistance. Third, reliable biomarkers are indispensable for patient stratification. Not all tumors exhibit equivalent dependence on glycolysis. Developing predictive models based on radiomics (e.g., heterogeneity parameters from ^18^F-FDG PET), liquid biopsies (metabolic gene variants or circulating metabolites), or molecular subtyping of tumor tissues will be essential to identify patient subgroups most likely to benefit from metabolic interventions, ultimately enabling true personalized cancer therapy.

In summary, integrating glycolytic-targeted therapy into the broader cancer treatment paradigm demands full-chain collaborative innovation—from molecular mechanisms and drug technology to clinical strategies. Only through such comprehensive efforts can the “Achilles’ heel” of tumor metabolism be transformed into tangible clinical benefits.

## Figures and Tables

**Table 1:** Enzymes involved in the canonical glycolytic pathway.

Step	Enzyme	Alternative Name	Reaction Catalyzed	Notes/Key Features
1	Hexokinase (Glucokinase in liver)	Hexokinase; Glucokinase (distinct isoform)	Glucose + ATP → Glucose-6-phosphate + ADP	Irreversible; traps glucose intracellularly; hexokinase feedback-inhibited by G6P, glucokinase is not
2	Phosphoglucose isomerase	Glucose-6-phosphate isomerase	Glucose-6-phosphate ⇌ Fructose-6-phosphate	Reversible isomerization
3	Phosphofructokinase-1 (PFK-1)	6-Phosphofructo-1-kinase	Fructose-6-phosphate + ATP → Fructose-1,6-bisphosphate + ADP	Major rate-limiting & committed step; activated by AMP/F-2,6-BP; inhibited by ATP/citrate
4	Aldolase	Fructose-bisphosphate aldolase	Fructose-1,6-bisphosphate → Dihydroxyacetone phosphate + Glyceraldehyde-3-phosphate	Cleaves 6-carbon sugar into two 3-carbon intermediates
5	Triose phosphate isomerase	Triosephosphate isomerase (TPI/TIM)	Dihydroxyacetone phosphate ⇌ Glyceraldehyde-3-phosphate	Rapid equilibrium; allows both trioses to enter glycolysis
6	Glyceraldehyde-3-phosphate dehydrogenase	GAPDH	Glyceraldehyde-3-phosphate + NAD^+^ + Pi → 1,3-Bisphosphoglycerate + NADH + H^+^	Oxidation + phosphorylation; produces NADH
7	Phosphoglycerate kinase	PGK	1,3-Bisphosphoglycerate + ADP → 3-Phosphoglycerate + ATP	Substrate-level phosphorylation; ATP production
8	Phosphoglycerate mutase	Phosphoglycerate mutase	3-Phosphoglycerate ⇌ 2-Phosphoglycerate	Intramolecular phosphate transfer; some isoforms require 2,3-BPG
9	Enolase	Phosphopyruvate hydratase; Enolase 1–3	2-Phosphoglycerate ⇌ Phosphoenolpyruvate + H_2_O	Dehydration forms high-energy intermediate
10	Pyruvate kinase	Pyruvate kinase (PKM1/PKM2/PKL/PKR)	Phosphoenolpyruvate + ADP → Pyruvate + ATP	Irreversible; substrate-level phosphorylation; PKM2 is dominant in cancer

**Table 3:** Glycolysis Inhibitors that Have Entered Clinical Studies and Reasons for Failure.

Inhibitor (Representative Drug)	Target	Clinical Status/Result	Main Reasons for Failure/Challenges	References
2-DG	HK2	Phase I/II, advanced solid tumors	Limited efficacy, high toxicity. The trial showed modest anti-tumor activity but significant adverse effects (e.g., hyperglycemia, QTc prolongation). The drug did not progress to later phases due to an unfavorable therapeutic window	[237]
3-BP	HK2, GAPDH, LDH	Preclinical/early clinical failure.	High toxicity: Non-selective alkylation caused damage to normal tissues. Delivery challenges: Poor solubility and rapid systemic clearance hindered effective tumor targeting.	[238]
AZD3965	MCT1	Phase I	Partial success, ongoing. Demonstrated target engagement (intracellular lactate accumulation) and manageable safety profile; however, efficacy as monotherapy was limited.	[113,239]
PFK158	PFKFB3	Phase I	Trial terminated. The study was discontinued due to insufficient anti-tumor activity and concerns about off-target effects.	[240,241]

## Data Availability

No new data were created or analyzed in this study. Data sharing is not applicable to this article.

## Abbreviations

The following abbreviations are used in this manuscript:

HK2	Hexokinase 2
PFK	Phosphofructokinase
PKM2	Pyruvate kinase M2
LDHA	Lactate dehydrogenase A
OXPHOS	Oxidative phosphorylation
TME	Tumor microenvironment
GLUTs	Glucose transporters
HK	Hexokinase
PK	Pyruvate kinase
LDH	Lactate dehydrogenase
PTMs	Post-translational modifications
HIF-1α	Hypoxia-Inducible Factor-1α
GLUT1	Glucose transporter type 1
PFKFB3	Enzyme 6-phosphofructo-2-kinase/fructose-2,6-bisphosphatase 3
PDK	Pyruvate dehydrogenase kinase
F-2,6-BP	Fructose-2,6-bisphosphate
PFK1	Phosphofructokinase 1
PGAM1	Phosphoglycerate mutase 1
PHD	Prolyl hydroxylase
G6P	Glucose-6-phosphate
HCC	Hepatocellular carcinoma
VDAC	Voltage-dependent anion channel
mPTP	Mitochondrial permeability transition pore
RCC	Renal cell carcinoma
ATC	Anaplastic thyroid carcinoma
PEP	Phosphoenolpyruvate
PPP	Pentose phosphate pathway
MDSCs	Myeloid-derived suppressor cells
PDH	Pyruvate dehydrogenase
DCA	Dichloroacetate
CAFs	Cancer-Associated Fibroblasts
TAMs	Tumor-Associated Macrophages
GPR81	G Protein-Coupled Receptor 81
CTLs	Cytotoxic T Lymphocytes
NK	Natural Killer
Tregs	Regulatory T cells
AMPK	AMP-Activated Protein Kinase
MCTs	Monocarboxylate transporters
2-DG	2-Deoxyglucose
ROS	Reactive oxygen species
3-BP	3-Bromopyruvate
DCs	Dendritic cells
MDR	Multi-drug resistance
ABC	ATP-binding cassette
RT	Radiotherapy
PDAC	Pancreatic ductal adenocarcinoma
GBM	Glioblastoma
TMZ	Temozolomide
HR	Homologous recombination repair
ICIs	Immune checkpoint inhibitors
PTT	Photothermal therapy
PDT	Photodynamic therapy
PDA	Polydopamine
NSCLC	Non-small-cell lung cancer
ADCC	Antibody-dependent cellular cytotoxicity
ENO2	Enolase 2
GAPDH	Glyceraldehyde-3-Phosphate Dehydrogenase
NEPC	Neuroendocrine prostate cancer
